# Hydrogels Augmented With Artificial Sweeteners can Inhibit Multidrug‐Resistant *Acinetobacter baumannii* Growth and Biofilm Formation While Demonstrating Safety in Pre‐Clinical Pilot Human Trials

**DOI:** 10.1002/adhm.202505777

**Published:** 2026-08-26

**Authors:** Jie Han, Mohamed Soliman, Bin Zhang, Dominika Krawiel, Ronan R. McCarthy

**Affiliations:** ^1^ Division of Biosciences Department of Life Sciences Antimicrobial Innovations Centre College of Health and Life Sciences Brunel University London Uxbridge UK; ^2^ National Biofilms Innovation Centre School of Biological Sciences Faculty of Environmental and Life Sciences University of Southampton Southampton UK; ^3^ Mechanical and Aerospace Engineering Department Brunel University London Uxbridge UK

**Keywords:** antibiotic resistance, artificial sweeteners, biofilm, hydrogel, wound

## Abstract

There is a critical need for novel therapeutic strategies to tackle multidrug‐resistant bacterial infections. Artificial sweeteners (AS) specifically acesulfame potassium, sodium saccharin, and sodium cyclamate, have recently demonstrated antimicrobial activity against multidrug‐resistant bacteria. In this study, polyvinyl alcohol (PVA)‐borate hydrogel is developed as a carrier for antimicrobial AS to combat wound infections. Through extensive optimization, we developed cytocompatible 8% AS‐loaded hydrogels with 3% PVA + 3% borax that reduced the viability of multidrug‐resistant *Acinetobacter baumannii* AB5075 by 99.9% colony‐forming unit enumeration following 1 h hydrogel treatment. Most currently available wound dressings have limited efficacy against bacterial biofilms, but we demonstrate that each of these sweeteners can attenuate *A. baumannii* and *Pseudomonas aeruginosa* dual‐microbial biofilms. Haemolysis and cell viability assays demonstrate excellent blood compatibility and non‐cytotoxic behavior of the sweetener‐loaded hydrogels. To further evaluate the clinical potential of these AS‐loaded hydrogels, we conducted a pilot Phase I clinical study with human volunteers focused on evaluating short‐term safety and irritancy. This study revealed that the dressings had no adverse effects on the volunteers. This research underscores the translational potential of hydrogels augmented with AS and their capacity to overcome many of the hurdles that typically lead to wound dressing failure.

## Graphical abstract

Using PVA–borax hydrogels as carriers for off‐the‐shelf artificial sweeteners to fight multidrug‐resistant *A. baumannii*. These soft, biocompatible hydrogels rapidly reduce bacterial viability within multidrug‐resistant *A. baumannii* biofilms by more than 99% within one hour and sustain antimicrobial activity over time, while demonstrating safety in both in vitro and pre‐clinical pilot human trials.

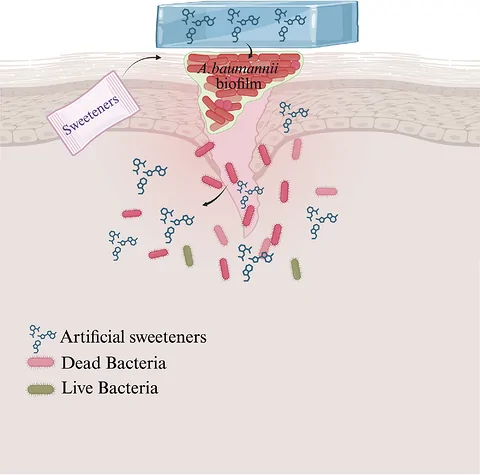

## 1 Introduction

Antimicrobial resistance (AMR) has become a public health crisis globally. It is estimated that deaths associated with AMR will reach 8.22 million annually by 2050 [1]. The misuse of antibiotics has accelerated the widespread dissemination of resistance among pathogens and created an urgent need for novel alternative antimicrobial strategies. Amongst the most recalcitrant pathogens is carbapenem‐resistant *A. baumannii*, which has been classified at the top of the World Health Organization's list of critical priority pathogens in urgent need of novel therapeutic interventions [2].

*A. baumannii* is an opportunistic nosocomial pathogen that is frequently associated with wound infections, ventilator‐associated pneumonia, bloodstream infections and urinary‐tract infections [3, 4, 5]. In 2021, *A. baumannii* accounted for 15.2% of all deaths attributable to AMR. The proportion of *A. baumannii*‐attributable deaths in patients aged five years and younger increased from 10.1% in 1990% to 13% in 2021 [1]. Resistance amongst these strains is a major problem, with up to 99% of the *A. baumannii* clinical isolates in some Mediterranean EU countries being multidrug‐resistant [6]. These rates are nearly 4 times higher than those observed for other Gram‐negative pathogens, such as *Pseudomonas aeruginosa* [7].

While its capacity to evolve or acquire resistance to antibiotics is one pillar supporting the pathogenic success of *A. baumannii*, its ability to form robust biofilms is another. Biofilms are groups of cells encased in an exopolysaccharide matrix which protects them from the rigors of disinfection, antibiotics and the host immune system. As a result of this capacity to form robust biofilms, *A. baumannii* is frequently associated with chronic infections where there are limited effective treatment options [8, 9].

Recent studies have demonstrated that artificial sweeteners (AS) have the ability to kill multidrug‐resistant bacteria through bulge mediated cell lysis and can attenuate *A. baumannii* virulence behavior by limiting motility and biofilm formation [10, 11]. Global transcriptomic analysis revealed that, saccharin (SACC) could trigger the down regulation of *A. baumannii* genes involved in pili and fimbriae biogenesis (*ABUW_0648, ABUW_1632, ABUW_1633, ABUW_2052, ABUW_2053, and ABUW_2310*), which are closely associated with bacterial adhesion and biofilm formation. Moreover, the genes encoding Csu pili (*ABUW_1487–1492*), as well as the biofilm‐associated protein (*bap*) gene (*ABUW_0916*), were significantly downregulated in the presence of Acesulfame K (Ace‐K) [10, 11]. In both studies, *A. baumannii* biofilm formation was inhibited in the presence of Ace‐K and SACC. Moreover, several *pil* genes (*pilN, pilO, pilA* in SACC, and *pilA, fimT, pilVWXY, pilGHIJL, pilZ, pilTU, pilBCD, and pilR* in Ace‐K) regulating twitching motility were down‐regulated, and this was further confirmed by the twitching motility assays. RNA‐Seq analysis of cells exposed to the sweeteners also revealed that several peptidoglycan amide and glycoside hydrolases were differentially expressed (including *ABUW_0928, ABUW_3204*, and *ABUW_4116*). This indicates that the disruption of the balance between peptidoglycan synthesis and degradation ultimately compromises cell envelope structural integrity, leading to the formation of bulges in the cell envelope that ultimately trigger lysis [10]. These changes in membrane integrity and permeability have also been shown to re‐sensitize *A. baumannii* to antibiotics and enhance the efficacy of carbapenems such as doripenem, imipenem, and meropenem in vitro. However, despite the remarkable in vitro antimicrobial activity of these sweeteners, a major challenge is how to effectively deliver them to the site of infection, particularly in wound environments where traditional methods may not provide sufficient localized concentrations reducing their effectiveness.

There are 6 types of wound dressings most commonly used in clinical settings: traditional dressings (gauze, cotton pads, and bandages), transparent film dressings, foam dressings, alginate dressings, hydrocolloid dressings and hydrogel dressings [12]. Hydrogels are three‐dimensional (3D) structured, hydrophilic, cross‐linked polymer networks [13]. They are widely used in medical applications for their ability to maintain a moist wound environment while also serving as vehicles for the controlled release of antimicrobial compounds [14]. The hydrophilic nature and biocompatibility of hydrogels make them ideal candidates for wound dressings, as they help maintain a moist healing environment, enable the delivery of antimicrobial agents to treat infections, and promote wound healing [15]. Hydrogels can be made from synthetic or natural polymers or both. Natural polymers such as collagen, gelatin, chitosan and hyaluronic acid provide exceptional biocompatibility and biodegradability. Nevertheless, they can exhibit comparatively low mechanical strength and can be degraded by enzymes, creating challenges for their use in therapeutic applications [16]. As a result, synthetic hydrogels are increasingly used in biomedical applications due to their excellent mechanical properties and low cost [17].

Polyvinyl alcohol (PVA)‐borate hydrogels have been widely studied for topical application due to their ability to retain moisture, conform to wound surfaces, enhanced mechanical properties, and capacity to release bioactive compounds in a controlled manner [18, 19]. The diol‐borate ester bonds in the PVA‐borate hydrogel improve its structural integrity, making it more robust for clinical applications. Through this study, we developed a series of highly potent AS‐loaded hydrogels that were optimized based on their swelling behavior, mechanical properties, gel porosity and antimicrobial efficacy against *A. baumannii* biofilms using in vitro models and ex vivo porcine skin burn wound models. We also demonstrated that these hydrogels were cytocompatible. To further demonstrate the translational potential of these hydrogels, we carried out a pilot Phase I human clinical study to evaluate the short‐term safety and irritancy of the optimized formulations on healthy volunteers. This trial revealed no adverse effects. This research provides a novel blueprint for effectively treating biofilm‐associated wound infections and addressing the critical issue of AMR.

## 2 Materials and Methods

### 2.1 Formulation of AS‐Loaded PVA‐Borate Hydrogel

AS either sodium saccharin, sodium cyclamate (99+%, Thermo scientific) or acesulfame potassium (99%, Sigma‐Aldrich), together with sodium tetraborate (99%, Alfa Aesar), and PVA (Mw = 115,000, 100% hydrolyzed, Sigma‐Aldrich) was prepared at final concentrations of 3%, 4%, 5%, 6%, and 8% (w/w), and sodium tetraborate was then added at final concentrations of 0.3%, 0.6%, 1%, 2%, 3%, 4%, and 5% (w/w). The deionized water was added to bring the final mass to 10 g. For example, a 3% PVA+0.6% sodium tetraborate + 8% SACC hydrogel contained 0.30 g PVA, 0.06 g sodium tetraborate, 0.8 g of SACC and 8.84 g deionized water before addition of AS. The beaker was heated in a water bath at 80°C with a watch glass on top for 3 h. The mixture was stirred every 1 h with a glass rod. After the hydrogel was obtained, it was transferred into custom‐made 3D‐printed molds to obtain a uniform shape. The diameter of the mound is 10 mm, and the height is 6.5 mm. The mound was 3D‐printed in a Mars 2 Pro 2K resin 3D printer (Elegoo). Freshly printed molds were UV‐cured in a Wash & Cure 2.0 chamber (Anycubic).

### 2.2 Hydrogel Zone of Inhibition

The suspension was adjusted to 0.5 McFarland using an overnight culture of *A. baumannii* AB5075 grown in Luria–Bertani (LB) broth (ThermoFisher) [20]. The Kirby‐Bauer disc diffusion method was used (Figure 1). Molded hydrogel (0.6 g) was plated on top of agar streaked with *A. baumannii* and then incubated for 24 h at 37°C. Three biological replicates of the zone of inhibition were measured by following: (d2‐d1) to avoid uneven distribution of the PVA‐borax hydrogel.

**FIGURE 1:**
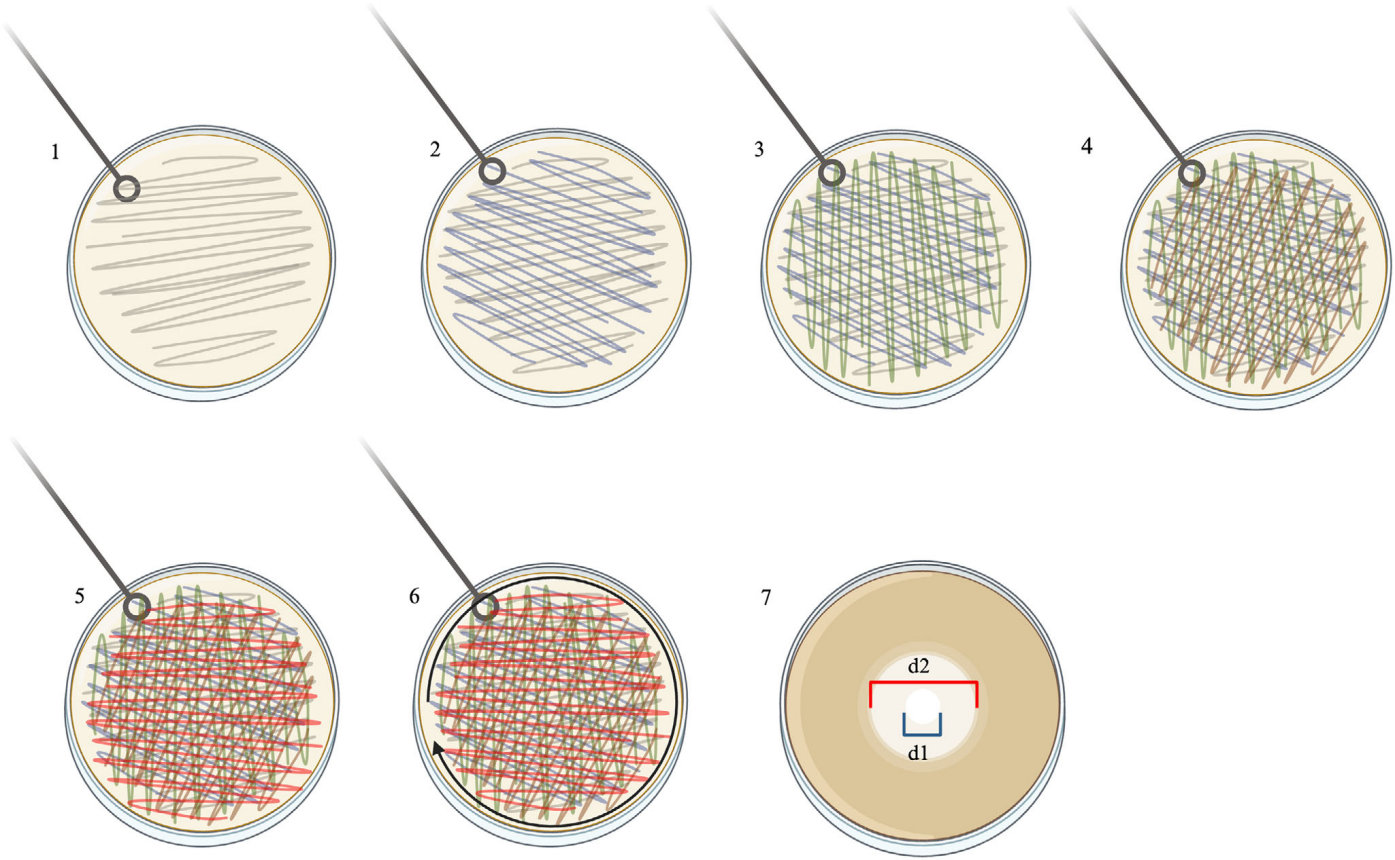
Illustration of *A. baumannii* AB5075 inoculation using the Kirby–Bauer disc diffusion method. The agar plate was swabbed five times (every 45°) in a linear pattern from 0° to 180°, followed by a sixth swab along the circumference to ensure even distribution at the edges. d1 indicates the diameter of the hydrogel, and d2 total diameter of the clear zone of inhibition surrounding the sample after incubation. Created in BioRender. Han, J. (2026) https://BioRender.com/wurdw3w.

### 2.3 Biofilm Assays and Scanning Electron Microscopy (SEM)

The OD_600_ of an *A. baumannii* AB5075 overnight culture was adjusted to 0.05 using sterile PBS. 1 mL of molten LB agar was applied to each well and allowed to solidify in a 12‐well plate. A 5 µL inoculum of *A. baumannii* AB5075 was applied to the agar surface and allowed to dry. The inoculated agar was then incubated for 3.5 h at 37°C and treated with either control hydrogel or AS‐loaded hydrogel (0.6 g) for 1 h. The treatment was then removed, and the biofilm and the bottom of the hydrogel were dispersed with 1 mL sterile PBS and washed for thrice. The collected PBS was serially diluted to 10^−7^ and plated to enumerate viable cells.

For the ex vivo assay, fresh porcine belly skin was purchased from Fine Food Specialist (London, UK). To mimic an acute burn wound, an array containing 20 steel pins was heated to 140°C for one hour and placed on the 20 cm^2^ pre‐cut skin for 60 s. The skin was cut into 1.5 cm^2^ pieces after the array was removed. Each side of the skin was sterilized under UV light for 1 h. The OD_600_ of an *A. baumannii* AB5075 overnight suspension was then adjusted to 0.05 using PBS. A 5 µL inoculum of AB5075 was applied to burnt skin and allowed to dry. The burnt skin with dried inoculum was incubated for 3.5 h in an incubator to form an early‐stage biofilm representing acute infection. The molded hydrogel (0.6 g) was applied on the top of the infected area. Also, Tegaderm Alginate Silver Dressing from MEDISAVE UK LTD (Dorset, UK) was cut into 1.5 cm^2^ pieces and placed on the infected skin. Then, the burnt skin was returned to the incubator for an additional 1 h. After incubation, the hydrogel or dressing was removed, and the skin was washed with 1 mL of sterile PBS and washed thrice. The collected PBS was serially diluted as before and plated to enumerate viable cells.

Scanning Electron Microscopy (SEM) was used to examine the biofilm formation of *A. baumannii* when it was exposed to AS‐Loaded PVA hydrogels. AS‐loaded hydrogel was placed into Falcon tubes with sterile glass slides. An *A. baumannii* AB5075 suspension adjusted to OD_600_ = 0.1 was then added to each tube, followed by incubation at 37°C for 24 h to allow biofilm formation on the glass surface. The glass slides with biofilm were fixed using 2.5% glutaraldehyde, 2% paraformaldehyde, and 0.15% alcian blue in 0.1 M phosphate buffer for 2 h, then treated with 1% osmium tetroxide in distilled water. The glass slides were dehydrated in graded ethanol, 50%, 75%, 90%, 100%, 100%, 30 min per step. Then, the glass slides were dried using 1:1 (Hexamethyldisilazane (HMDS): ethanol) and twice in 99.9% HMDS, 15 min per step [21]. Finally, samples were gold‐coated and observed by SEM (JEOL IT200, JEOL, USA).

### 2.4 Antimicrobial Time‐Kill Assay and Drug Release Profile

Control hydrogels and AS‐loaded hydrogels were prepared, weighed (0.6 g), and each gel was placed into a well of sterile 24‐well plates and incubated for 15 min at 37°C to ensure that the hydrogel fully covered the bottom of each well. A 1 mL suspension of *A. baumannii* AB5075, adjusted to an OD_600_ of 0.5 from an overnight culture, was then added to each hydrogel, and the plates were incubated at 37°C. This setup was repeated for each time point (0, 2, 4, 6, 8, and 24 h), and at each time point, the bacterial suspension from each well was collected, serially diluted, and plated to enumerate viable cells.

The in vitro drug‐release behavior of the drug‐loaded hydrogel was evaluated using a modified pharmaceutical dissolution test. The hydrogels were placed in 25 mL of PBS at pH 7.4 and pH 5.5 at 100 rpm and 37°C in a KS 3000 control incubator shaker (IKA, Staufen, Germany). At predetermined time intervals, 2 mL samples were withdrawn and replaced with an equal volume of fresh release medium. The absorbance of the collected samples was measured using UV–Vis spectrophotometry (VWR) at λ = 270 nm, the wavelength of maximum absorption for SACC. Drug content was calculated based on the established calibration curve. All experiments were conducted in triplicate.

### 2.5 A. *baumannii* AB5075 and *P. aeruginosa* PA14 Mixed Culture Assays

In a 12‐well plate, 1 mL of molten LB agar was applied to each well and allowed to solidify. The OD_600_ was adjusted to 0.05 for each overnight *A. baumannii* AB5075 and *Pseudomonas aeruginosa (P. aeruginosa)* PA14 suspension culture using sterile PBS. A 500 µL of *A. baumannii* inoculum and a 500 µL of *P. aeruginosa* inoculum were transferred into a tube. A 5 µL inoculum of *A. baumannii* and *P. aeruginosa* mixture was applied to the surface of the agar and allowed to dry. The agar was incubated for 3.5 h at 37°C to form a biofilm. The molded hydrogel was applied to the biofilm. Then, the agar was transferred back to the incubator for an extra 1 h. The hydrogel was removed, and the agar surface was washed with 1 mL of sterile PBS to resuspend the biofilm. The washing process was repeated 3 times, diluting the collected PBS and enumerating viable cells. To enumerate viable *P. aeruginosa* PA14 cells, *Pseudomonas* isolation agar (Sigma‐Aldrich) was used. To enumerate viable cells of *A. baumannii* AB5075, gentamicin was added to LB agar. The final gentamicin concentration in the agar was 20 µg/mL.

### 2.6 Haemolysis Assays Cytocompatibility Assessment

To evaluate haemolysis, erythrocytes were collected from 10 mL of defibrinated horse blood (TCS Bioscience, UK) by centrifugation of whole blood at 3000 rpm for 10 min, followed by 3 washes with 10 mL PBS to isolate erythrocytes. Hydrogel extracts were prepared by incubating 0.2 g of hydrogels in 1 mL PBS at 37°C for 24 h. For the haemolysis test, 1% v/v erythrocytes were mixed with hydrogel extracts and incubated at room temperature for 1 h, with deionized water as the positive control and PBS as the negative control. The samples were centrifuged, and the 100 µL supernatant was collected and transferred to a 96‐well plate. Haemoglobin release was quantified by measuring absorbance at 541 nm using a microplate reader. The haemolysis rate was calculated by using the following equation [22]:

$$\mathrm{Haemolysis}\mathrm{rate}\left(\%\right)=\left(OD_{\exp}-OD_{\mathrm{neg}}\right)/\left(OD_{\mathrm{pos}}-OD_{\mathrm{neg}}\right)\times 100\%$$

where OD_exp,_ OD_neg_, and OD_pos_ represent the OD values for the experimental, negative, and positive groups.

Hydrogel cytocompatibility was assessed using an MTT assay with dermal fibroblasts (2DD cell line) previously isolated by Bridger et al. [23]. In 24‐well plates, cells (5 × 10^4^ /well) were added in DMEM and supplemented with 10% FBS, 2 mM L‐glutamine, and 1% penicillin/streptomycin, with a final volume of 500 µL per well. The cells were then incubated at 37°C with 5% CO_2_ for 24 h. Nonadherent cells were removed by washing twice with PBS, and fresh medium or hydrogel extract medium (500 µL) was added to fibroblast‐seeded wells, followed by incubation for 24 h. Following treatment, the cells were washed twice with PBS.

Three experimental groups were included: untreated cells (blank group), cells treated with PVA hydrogel extract (control group), and cells treated with AS‐loaded hydrogel extracts (experimental group). Following treatment, cells were washed twice with PBS and incubated with 500 µL MTT solution (0.5 mg/mL in PBS) for 3 h. The supernatant was subsequently removed, and the resulting formazan crystals were dissolved in 750 µL dimethyl sulfoxide (DMSO). Optical density was measured at 570 nm using a microplate reader. Cell viability was expressed as the percentage absorbance relative to untreated control cells.

### 2.7 Degree of Swelling and Weight Loss of Hydrogels

The degree of swelling was determined by the maximum amount of liquid that hydrogels could absorb. In this experiment, the pH of the PBS solution was set at 7.4 to mimic wound site exudate. Two grams of AS‐loaded PVA hydrogels were freeze‐dried overnight, and their final weights were measured thrice until no further weight change was observed. The dried hydrogels were then immersed in pH‐adjusted PBS at 37°C for 48 h, and their weight was measured at 0, 3, 6, 9, 24, and 48 h. The degree of swelling (DS) was calculated by using the formula below. The *m_i_* is the weight at a specific hour, and *m_0_* is the weight of the dry hydrogel at 0 h.

$$\mathrm{SD}=\frac{m_{i}-m_{0}}{m_{0}}\times 100\%$$

The weight loss (WL) observed in the hydrogel can be an indicator for its degradation. To determine this, the swollen hydrogel was freeze‐dried again at the end of the swelling test. The dry weight of the hydrogel was then measured, and the WL was calculated. The *W_i_* is the initial dry weight, and *W_d_* is the weight of the re‐dried hydrogel after 48 h of soaking.

$$\mathrm{WL}=\frac{W_{i}-W_{d}}{W_{i}}\times 100\%$$

### 2.8 Hydrogel Morphology

SEM was used to characterize the morphological properties of the PVA hydrogels. Samples were snap‐frozen in liquid nitrogen, freeze‐dried, mounted onto carbon discs, then gold‐coated and analyzed using SEM (Jeol IT200, JEOL, USA). SEM images at magnifications of 250x were captured to examine the pore size and cross‐linking density of the hydrogels after freeze‐drying. The pore size of the hydrogels was then measured using ImageJ software. For each hydrogel, three images were analyzed, each covering an area of 512.5 µm x 383.9 µm.

### 2.9 Rheology

Rheological properties of the hydrogels were measured using a TA Instruments Discovery Core Rheometer (TA Instruments, USA) with a 40 mm parallel plate geometry. A solvent trap was applied to reduce water evaporation during testing. The amplitude sweep was conducted to assess the hydrogel's linear viscoelasticity, and the frequency sweep was performed to determine the gel's stiffness. The strain range of the amplitude sweep was 0.01%–100% at 1 Hz. The frequency sweep was measured between 0.1–100 rad/s at 0.1% strain. All measurements were performed at 37°C to mimic physiological conditions. All tests were conducted in triplicate.

### 2.10 Attenuated Total Reflectance–Fourier Transform Infrared Spectroscopy (ATR‐FTIR) and X‐Ray Diffraction (XRD)

Fourier Transform Infrared (FTIR) spectroscopy was measured by using a Spectrum One (PerkinElmer, USA) and used to investigate the chemical interaction between the functional groups in the cross‐linked structure of the hydrogels. FTIR spectra were used to study the structural changes in PVA hydrogel loaded with different AS. Samples were compared for the identification of any shifts or variations in the characteristic peaks, which could reflect changes in the bonding pattern and interaction between PVA and the sweetener molecules. Measurements were taken in a wavelength region of 600–4000 cm^−^^1^ with 10 scans. The crystalline profiles of AS‐Loaded hydrogels were studied by using an x‐Ray Diffractometer (D8 Advance, Bruker). The x‐ray source used Cu‐Kα radiation at 40 kV and 40 mA;2θ was from 5 to 100.

### 2.11 Pilot Phase 1 Clinical Study in Humans

To assess the short‐term dermal compatibility of AS‐Loaded PVA‐borax hydrogel, healthy human volunteers were monitored for immediate skin reactions and potential adverse effects following topical application. Before the pilot study, the pH of hydrogels was measured. Hydrogels (3 replicates per hydrogel) were immersed in water for 24 h, and the pH was measured with a pH meter. A Pilot Phase 1 clinical study in humans. This experiment protocol was accepted by the University Research Ethics Committee (UREC) at Brunel University London, UK. The Ethical approval number is 47765‐MHR‐Nov/2024‐53276‐3 (Figure S5). A total of 12 healthy adult volunteers aged 18–65 years were eligible and recruited from Brunel University of London for this study. All participants had no irritated or abraded skin at the measurement sites and had not suffered from any skin conditions, including eczema and dermatitis. The participant's skin condition was assessed by a self‐questionnaire and the researcher. Four skin sites were chosen: the lower left forearm (LL), the upper left arm (UL), the lower right forearm (LR), and the upper right arm (UR). Each site had an area of skin 1.5 x 3 cm tested. On each site, 3 replicates of 0.2 g hydrogel were applied and monitored. A film dressing (TEGADERM Film Dressings, 3 M) was applied over the hydrogels. All participants were observed at 1, 3, 6, 9, and 24 h.

### 2.12 Statistical Analysis

Data are expressed as mean ± standard deviation (SD). Statistical analyses were performed using GraphPad Prism and Origin Pro. Two‐way ANOVA with Tukey correction and one‐way ANOVA with Dunnett's correction were used. Statistical significance was established at *p*‐values of < 0.05 (*), 0.01 (**), 0.001 (***) or 0.0001 (****).

## 3 Results

### 3.1 Formulation of AS Loaded PVA‐Borate Hydrogel

The AS Ace‐K, SACC, and CYC have displayed promising therapeutic potential when administered in solution in in vitro assays [10, 11]. A major roadblock to the translation of novel antimicrobials is effective delivery solutions that can be applied in vivo. To assess if these sweeteners could be integrated into hydrogels and maintain their antimicrobial activity, different formulations were screened to optimize the structural stability of the AS‐loaded PVA‐borax hydrogels. Initial tests showed that the addition of 3% (w/w) AS led to phase separation between the AS, water, and gel matrix, leading to turbidity and instability in several formulations. To solve this, a series of hydrogels were prepared by combining 3%, 4%, 5%, 6%, and 8% (w/w) PVA with 0.3%, 0.6%, 1%, 2%, 3%, 4%, and 5% (w/w) sodium tetraborate (borax), each with 4%, 6%, and 8% (w/w) of AS. When 4% (w/w) AS was added, hydrogels made with 3%, 4%, and 5% (w/w) PVA and 2%–5% (w/w) borax remained structurally stable. However, when the AS concentration was increased to 6% (w/w), only 3% PVA combined with 2%–5% borax produced stable hydrogels. The 4% PVA also formed hydrogels under these conditions. However, the hydrogel began to change from transparent to milky in appearance, and phase separation occurred within 30 min. At 8% (w/w) AS concentration, only the 3% PVA with 3%–5% borax resulted in structurally stable gels, while all other tested formulations failed to form hydrogels or exhibited phase separation. Based on these results, the formulation of 3% PVA and 3% borax was selected for all subsequent experiments, as it consistently supported gel formation across all AS concentrations tested (4%, 6%, and 8%) while maintaining adequate mechanical and visual stability (Table 1).

**TABLE 1:** Formulation screening of AS‐loaded PVA–Borax hydrogels and their structural stability.

PVA (% w/w)	AS (% w/w)	Borax (% w/w)	Gel formation
3, 4, 5, 6, 8	4	0.3%, 0.6%, 1%	Phase separation, low elastic, brittle gel
3, 4, 5	4	2%–5%	Stable gel
6,8	4	2%–5%	Phase separation, low elastic, brittle gel
3	6	2%–5%	Stable gel
4, 5	6	2%–5%	Phase separation, low elastic, brittle gel
3	8	3%–5%	Stable gel
3	8	2%	Phase separation, low elastic, brittle gel

### 3.2 AS‐Loaded Hydrogels Showed Antimicrobial Activity Against *A. baumannii* in a Disk Diffusion Assay

After developing the AS hydrogel formulae, the hydrogels were tested for their antimicrobial properties against a multidrug‐resistant clinical isolate of *A. baumannii* using the disk diffusion method. Ace‐K, SACC, and CYC‐loaded hydrogels showed significant dose‐dependent antimicrobial properties when compared to the control hydrogel (borate‐based, without sweeteners). As shown in Figure 2, the higher the concentration of AS, the more antimicrobial activity. The average zone of inhibition (ZOI) increased with Ace‐K loading. Hydrogels containing 1% and 2% Ace‐K exhibited mean ZOIs of 4.83 ± 0.29 mm and 5.33 ± 0.29 mm, respectively, compared with 3.50 mm for the control hydrogels. The control gel also had a ZOI, which is in agreement with the known antiseptic effect of borate [24]. There was a substantial increase in ZOI with hydrogels loaded with 4%, 6% and 8% Ace‐K, showing an average ZOI of 5.5 ± 0, 6.17 ± 0.29 and 8 ± 0.5 mm. The ZOI for the 1% SACC hydrogel was 4.16 mm, for the 2% SACC hydrogel it was 5 mm, and for the 4% SACC hydrogel it was 5.17 mm. The 6% SACC‐loaded hydrogel exhibited an average ZOI of 5.67 ± 0.29 mm, while the 8% SACC‐loaded hydrogel showed an average ZOI of 7 ± 0.5 mm. The CYC‐loaded hydrogels were also evaluated and showed concentration‐dependent antimicrobial effects like to other developed AS‐loaded hydrogels. CYC‐loaded hydrogels start to show significant antimicrobial activity compared with the control gels beginning at concentrations of 4%. Notably, the antimicrobial efficacy increased significantly at CYC concentrations of 4%, 6%, and 8%, with average ZOI values of 5.83 ± 0.29 mm, 7.17 ± 1.03 mm, and 8.83 ± 1.26 mm. All three AS‐loaded hydrogels at 6% and 8% concentration showed the highest antimicrobial activity. Therefore, they were selected for further study.

**FIGURE 2:**
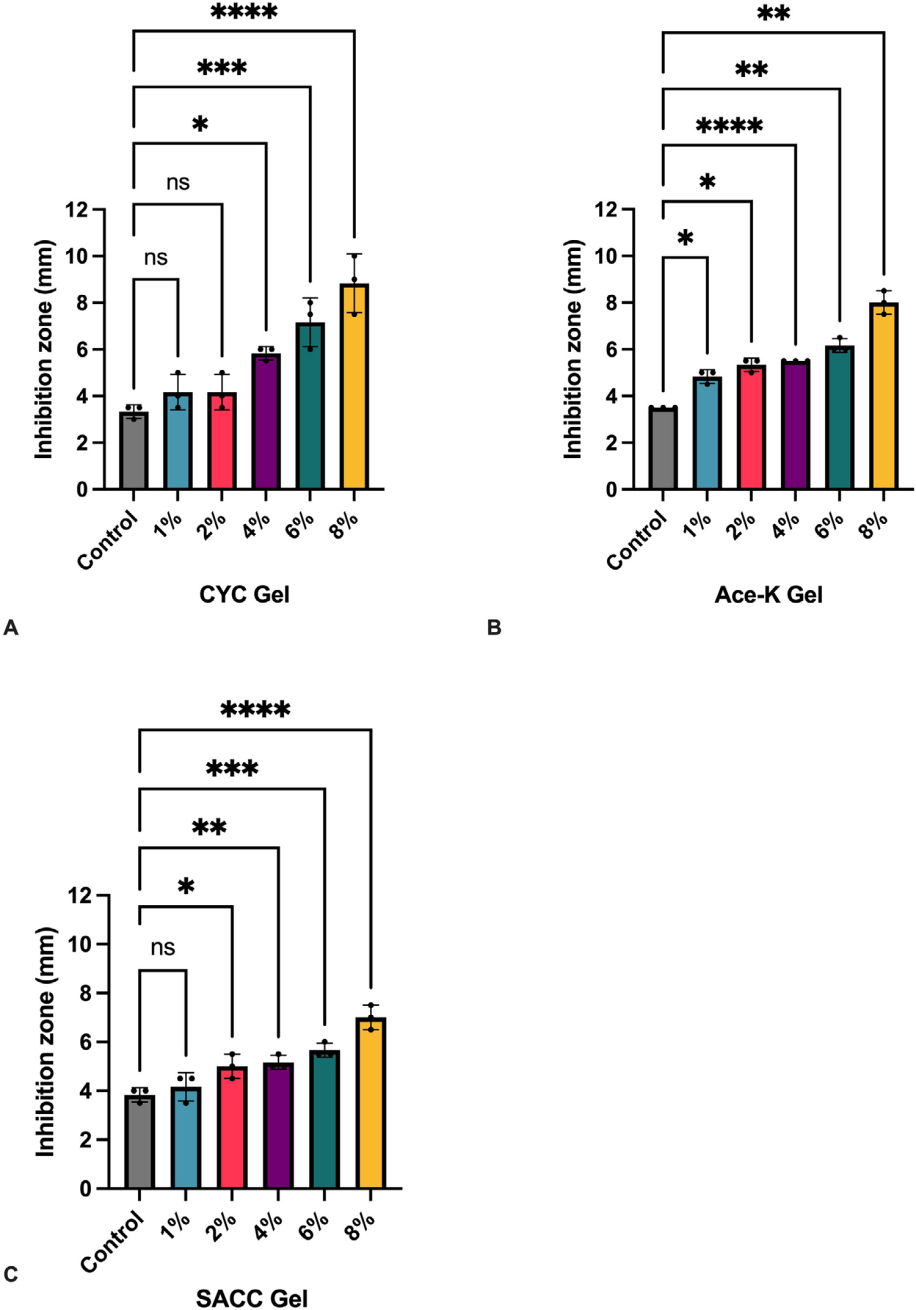
Antimicrobial activity of hydrogels loaded with (A) Ace‐K, (B) SACC and (C) CYC against *A. baumannii*, assessed by disk diffusion assay. The antimicrobial activity increases proportionally with the AS concentration. Among all AS‐loaded hydrogel, CYC hydrogel showed the largest ZOI at 8%. The ZOI increased significantly with higher concentrations of Ace‐K (≥4%), SACC (≥6%), and CYC (≥4%), compared to control hydrogels. Data represent the mean ± SD from three biological replicates. Statistical significance was determined by ordinary one‐way ANOVA (*ns*, *p* > 0.05; *, *p* <0.05; **, *p* <0.01; ***, *p* <0.001; ****, *p* < 0.0001).

### 3.3 AS‐Loaded Hydrogels Exhibit Substantial Antibiofilm Activity against *A. baumannii* In Vitro

Many antimicrobials show limited efficacy against biofilms. Bacteria in a biofilm can require antibiotic concentrations that are 10 to 1,000 times higher for eradication than their planktonic counterparts [25]. These sweeteners have demonstrated antibiofilm potential previously via the dysregulation of key genes linked to biofilm formation [10]. To evaluate if this antibiofilm potential was maintained when the AS were formulated as part of a hydrogel, we used the colony biofilm agar model, and we initially checked early‐stage biofilm (3.5 h) [10]. The control hydrogel (borate‐based, without sweeteners) demonstrated a reduction (∼ 1.98 log CFU; p <0.01) in viable cells of *A. baumannii* after 1 h treatment when compared with the untreated group (Figure 2A). Notably, loading hydrogels with AS led to significantly enhanced biofilm eradication. The 8% Ace‐K hydrogel achieved ∼4.00 log reduction in CFUs, which corresponds to more than a 99.99% reduction in viable biofilm cells. SACC and CYC hydrogels demonstrated even higher efficacy, with reductions of 5.44 and 5.67 log CFU, respectively, indicating near‐complete biofilm eradication after just 1 h of treatment. These results highlight the potent and rapid antibiofilm activity of AS‐loaded hydrogels against early‐stage biofilms.

In the 24‐hour established biofilm model (Figure 3B–D), AS‐loaded hydrogels showed a significant time‐dependent biofilm reduction over a 24 h treatment period compared to the control gel. The control gel only showed ∼1.77 log reduction in CFU/biofilm without further reduction observed up to 24 h when compared with the untreated group, showing the limited effect of the control gel. In contrast, the Ace‐K hydrogel produced a ∼1.74 log reduction at 2 h, with continued decline in biofilm burden over time, reaching ∼2.51 log at 8 h and ∼4.48 log at 24 h when compared with the untreated group. Similarly, the SACC hydrogel caused a ∼1.84 log reduction at 2 h, improving to ∼2.82 log and ∼4.79 log by 8 h and 24 h. The CYC hydrogel also showed ∼1.07 and 2 log reduction in CFU within 2 and 4 h, culminating in 3.94 log reduction after 24 h of treatment. These results confirm a sustained and potent antibiofilm effect likely due to the controlled release of AS from the hydrogel matrix. To visualize these antibiofilm and antibacterial effects, we performed SEM on established biofilms treated for 24 h with the AS loaded hydrogels. The SEM image of the AS‐loaded hydrogel reveals significant antibacterial and antibiofilm activity (Figure 3E). Compared to the control and untreated groups, the AS‐loaded hydrogel exhibited significantly reduced *A. baumannii* biofilms. Moreover, CYC resulted in the smallest reduction in *A. baumannii*. This finding aligns with Figure 3B–D and further confirms the antibiofilm potential of these AS‐loaded hydrogels. Additional SEM images are shown in the Supporting Information (Figure S1).

**FIGURE 3:**
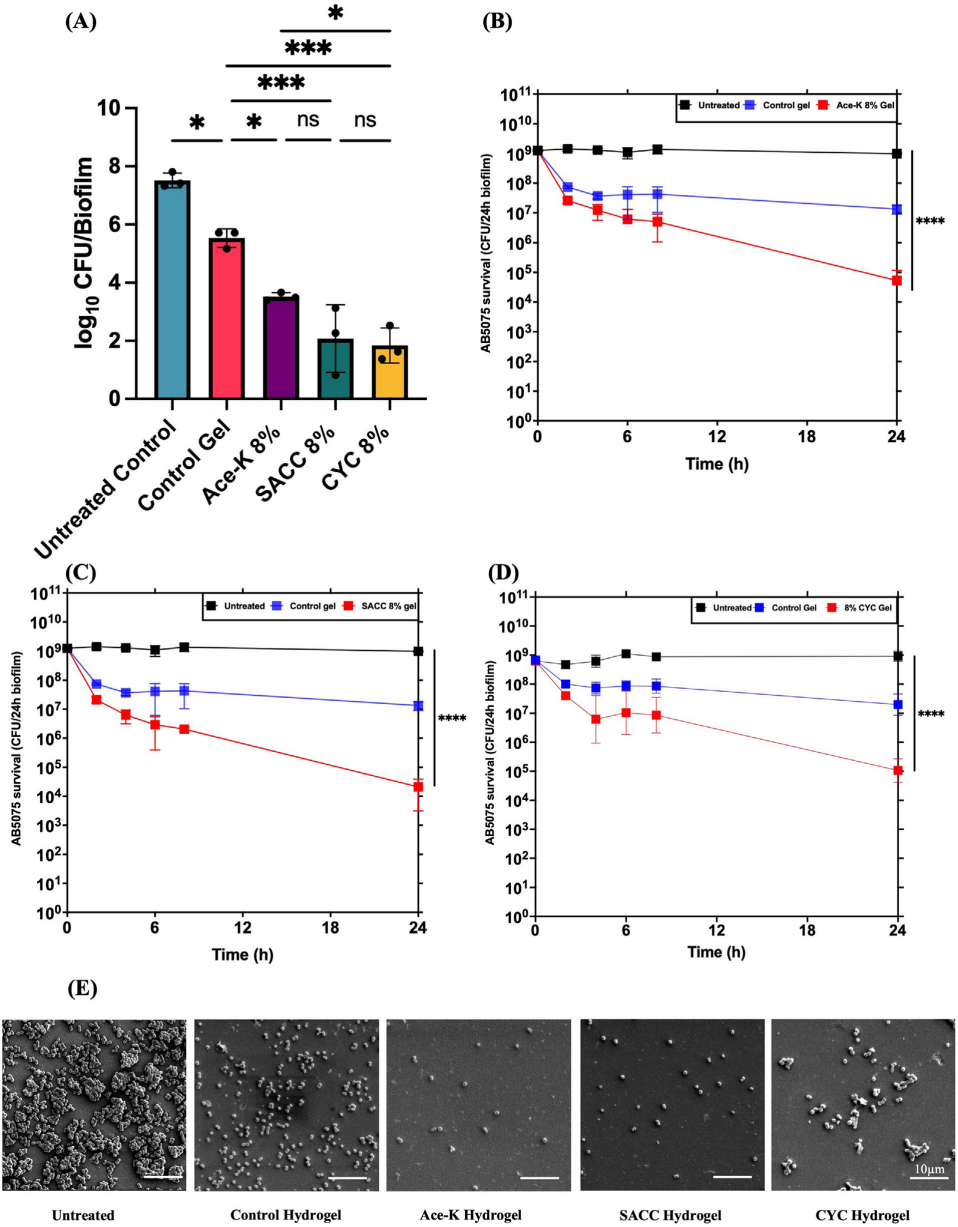
Antibiofilm activity of AS‐loaded hydrogels against *A. baumannii* in vitro. (A) Viable cell counts (log_10_ CFU/biofilm) of 3.5 h early established *A. baumannii* biofilms following 1 h treatment with either control hydrogel or 8% AS‐loaded hydrogels. Sweetener hydrogels significantly reduced the biofilm compared to the control hydrogel. (B‐D) Time‐kill kinetics of mature *A. baumannii* biofilms treated with AS‐loaded hydrogels (B) 8% Ace‐K, (C) 8% SACC, and (D) 8% CYC. CFUs were quantified at 2, 4, 6, 8, and 24 h post‐treatment. SACC and Ace‐K hydrogels exhibited sustained and significant reductions in viable biofilm cells over 24 h compared with the control hydrogel. (E) Representative SEM image of *A. baumannii* with 24‐hour treatment AS‐Loaded PVA hydrogel, Control hydrogel, and Untreated Control. Data are presented as mean ± SD (n = 3). One‐way ANOVA and two‐way ANOVA with multiple comparisons were performed to assess statistical significance. (ns >0.05, * *p*‐ value < 0.05, ** *p*‐value < 0.01, ***p‐value <0.001, **** *p* value < 0.0001).

### 3.4 AS‐Loaded Hydrogels Outperform Commercial Antimicrobial Silver Alginate Hydrogels in an *A. baumannii* Early Biofilm Ex Vivo Model

To further validate the antibiofilm potential of AS‐loaded hydrogels in a clinically relevant setting, a porcine ex vivo burn wound infection model was employed to compare their efficacy against a commercially available silver dressing [10]. After 1 h of treatment, the control gel resulted in a slight, yet nonsignificant reduction in *A. baumannii* biofilm burden indicating its limited activity in a short exposure time (Figure 4). In contrast, all AS‐loaded hydrogels demonstrated significantly enhanced antibiofilm activity when compared with the control gel. The Ace‐K hydrogel achieved a reduction in CFU/biofilm by ∼1.48 log, while SACC‐ and CYC‐loaded hydrogels followed a similar pattern of the in vitro assay and demonstrated significant antibiofilm activity, reducing the bacterial cell numbers by ∼2.64 and ∼2.76 log in biofilm when compared with the control gel and ∼2.37, ∼3.53, and ∼3.65 log when compared with the untreated group. These findings indicate a strong, rapid disruption of early biofilms by our developed AS hydrogels, with SACC and CYC showing the most potent effects, achieving more than 99% biofilm reduction within 1 h. Surprisingly, the commercial silver dressing, a widely used standard for topical antimicrobial treatment, failed to show any significant effect against the *A. baumannii* biofilm during short‐term exposure, highlighting the limited efficacy of current frontline treatments against *A. baumannii* AB5075 biofilms. Taken together, these results demonstrate that the AS‐loaded hydrogels not only possess rapid and potent antibiofilm properties but also significantly outperform the commercial silver dressing under short contact conditions.

**FIGURE 4:**
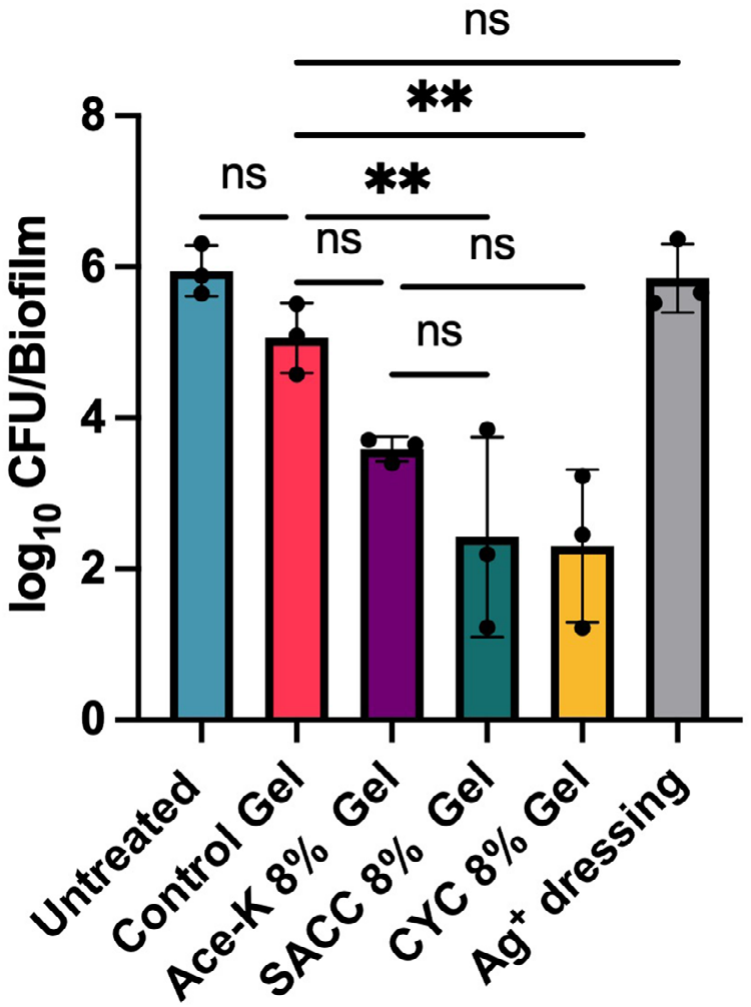
Antibiofilm activity of AS‐loaded hydrogels against *A. baumannii* in ex vivo porcine skin burn wound model. Viable cell counts (log_10_ CFU/biofilm) of 3.5 h *A. baumannii* biofilms following 1 h treatment with either control hydrogel, 8% AS‐loaded hydrogels or silver dressing. Sweetener hydrogels significantly reduced the biofilm compared to the control hydrogel and silver dressing (n = 3 ± SD). One‐way ANOVA with multiple comparisons was performed to assess statistical significance. (ns >0.05, ** p <0.01).

### 3.5 AS‐Loaded Hydrogels Exhibit Time‐Dependent Antimicrobial Activity Against *A. baumannii* In Vitro

For hydrogel wound dressing applications, the treatment interval typically ranges from 1 to 3 days [26]. A slow release of the AS profile can prolong its activity at the wound site and help prevent bacterial contamination during the treatment window, but also be more effective against established biofilms [26, 27]. In earlier results, AS hydrogels showed time‐dependent antimicrobial activity against *A. baumannii*. Moreover, this effect was more pronounced when higher amounts of AS were loaded, as more AS was released from the gel matrix (Figure 3). Previous work has demonstrated that these sweeteners are bactericidal rather than bacteriostatic. To determine whether slow‐release kinetics at concentrations sufficient to mediate this bactericidal activity could be achieved from the AS‐loaded hydrogels, the hydrogels were submersed in PBS containing a known concentration of bacterial cells. PBS was used to limit the bacterial proliferation while maintaining cell viability. This approach would ensure any changes in bacterial counts were primarily attributable to the bactericidal activity of the AS.

After 24 h, the bacterial cell count in the control gel was reduced by around 1.03 log CFU/mL when compared with the untreated group. However, all AS‐loaded hydrogels resulted in a significant reduction in CFU/mL. Among all AS‐loaded hydrogels, both Ace‐K and CYC demonstrated the most potent antimicrobial activity. After 24 h, hydrogels loaded with 6% AS showed an average bacterial reduction of ∼5.27 log in CFU count compared to the growth control. The 6% CYC‐loaded hydrogel exhibited the highest reduction of ∼6.26 log CFU/mL, followed by the 6% Ace‐K‐loaded hydrogel with a ∼5.47 log CFU/mL of reduction and the 6% SACC‐loaded hydrogel with a ∼4.08 log of reduction in CFU count (Figure 5A, C). Increasing the AS concentration further enhanced inhibition, indicating greater AS release over time. Hydrogels loaded with 8% AS achieved an average reduction in CFU count of ∼6.13 log units compared to the growth control. The 8% CYC‐loaded hydrogel again demonstrated a reduction of ∼6.42 log in CFU/mL after 24 h, while the 8% Ace‐K‐loaded hydrogel achieved a reduction of ∼6.41 log. The 8% SACC‐loaded hydrogel reduced bacterial growth by ∼5.56 log. In contrast, the control hydrogel showed a nonsignificant reduction in CFU count (Figure 5B, D). These results highlight the dose‐dependent antimicrobial activity of AS‐loaded hydrogels, with consistent release over time. The CYC hydrogel provides the strongest inhibition, followed by the Ace‐K and SACC hydrogels.

**FIGURE 5:**
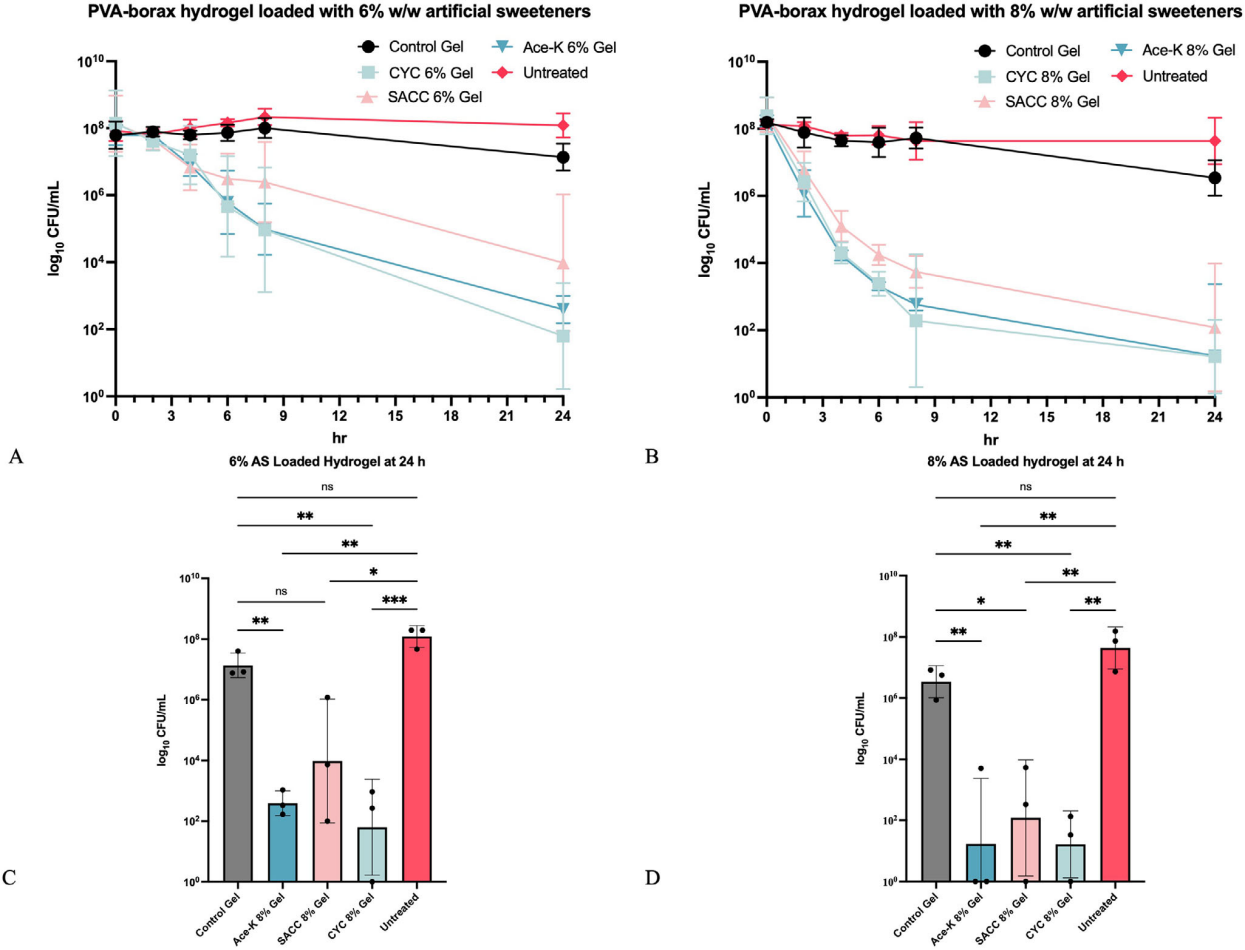
AS Hydrogel Effectiveness over 24 h: (A, B) Antimicrobial profile of AS‐loaded hydrogels at different AS concentrations. (C, D) Antimicrobial activity of 6% and 8% AS‐Loaded Hydrogels Against *A. baumannii* at 24 H. Data demonstrate the mean of three biological replicates. Error bars represent standard deviation (SD). Statistical analysis was performed by one‐way ANOVA between the control and treated samples. (ns>0.05, * *p*‐ value < 0.05, ** *p* value *<* 0.01, *** *p* value *<* 0.001).

The SACC loaded‐h32ydrogel release profile was studied and showed a slow release at both pH 7.4 and pH 5.5 (Figure S2). This data indicates that SACC reaches the fully released state around 4–6 h, which aligns with the time‐kill assay shown in Figure 4. This pH range covers the optimum growth pH for wound bacteria [28, 29].

### 3.6 AS‐Loaded Hydrogels Reduce *A. baumannii* and *P. aleruginosa* Dual‐Species Biofilms

In a clinical setting, biofilms are rarely found as monospecies, instead they typically comprise of multiple different microorganisms in a polymicrobial biofilm [8]. These polymicrobial biofilms offer even greater protection against the host immune system and antimicrobials, which are more difficult to treat [30, 31]. Apart from *A. baumannii*, *P. aeruginosa* is a major contributor to chronic wound infections, with prevalence rates ranging from 20% to 75%, making it a key target for treatment [32, 33]. To assess the efficacy of AS‐loaded hydrogels against a dual‐species biofilm, we utilized an in vitro mixed‐culture biofilm model combining *A. baumannii* and *P. aeruginosa*, followed by plating on selective media after treatment, which allowed growth of only *A. baumannii* or *P. aeruginosa*. As shown in Figure 5, all 8% AS‐loaded hydrogels demonstrated significant dual‐species biofilm inhibition. For *A. baumannii* (Figure 6A), the CYC‐loaded hydrogel had the most potent impact on bacterial survival, reducing *A. baumannii* viable cells by ∼4.46 log. SACC and Ace‐K‐loaded hydrogel demonstrated ∼3.56 and ∼3.75 log reductions in *A. baumannii* viable cells. All AS‐loaded hydrogels demonstrated more than a 3‐log reduction compared with the untreated group, while the control hydrogel showed a 1.63‐log reduction. This means that all AS‐loaded hydrogels achieved 99.9% inhibition.

**FIGURE 6:**
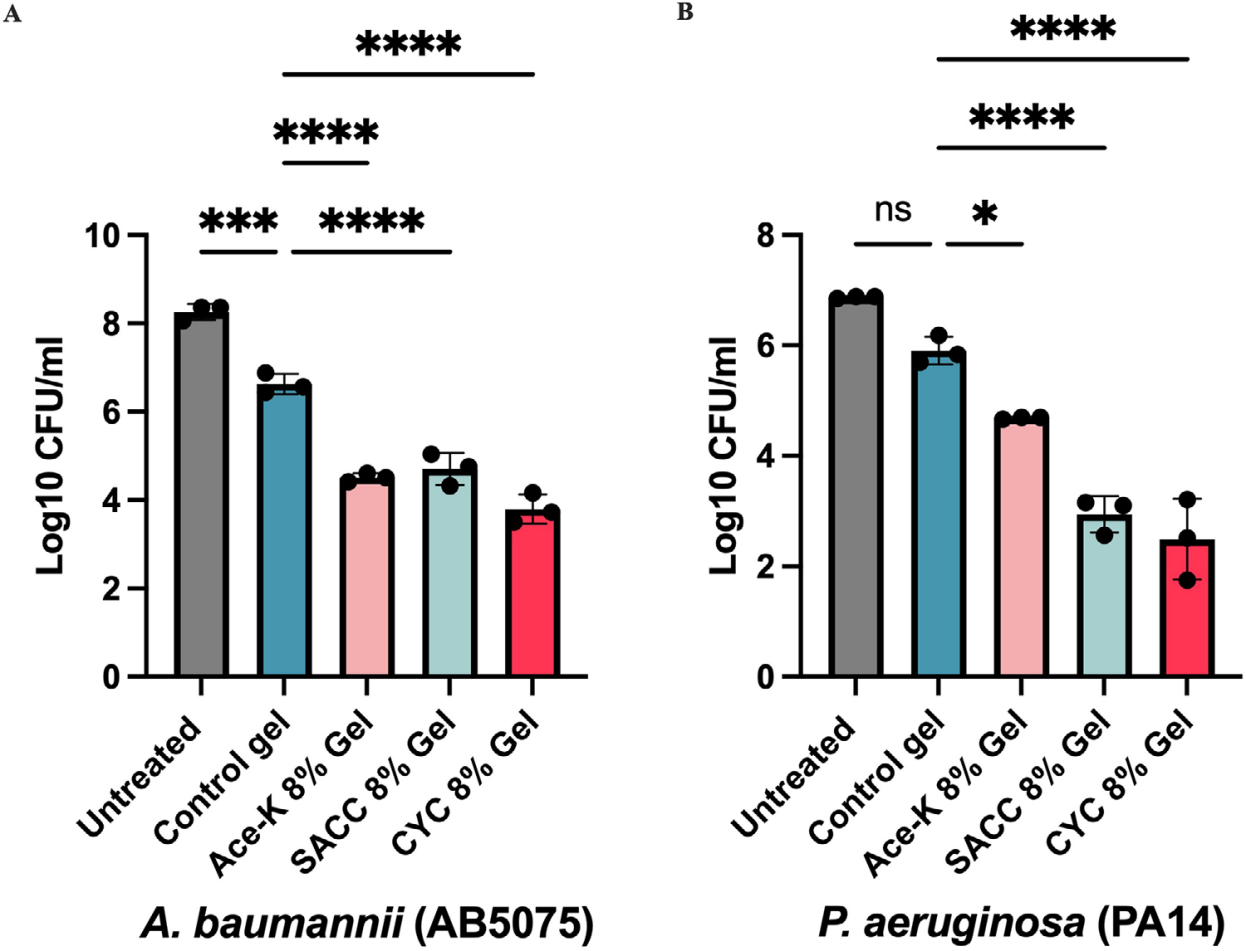
Antimicrobial activity of AS‐loaded hydrogels against mixed infection biofilm in vitro. (A) Viable cell counts (Log_10_ CFU/mL) of *A. baumannii* AB5075 after treatment with AS‐loaded hydrogel. (B) Viable cell counts (Log_10_ CFU/mL) of *P. aeruginosa* PA14 after treatment with AS‐loaded hydrogel. All AS‐loaded hydrogels reduce bacterial viability compared to untreated and control hydrogel groups. The CYC‐loaded hydrogels demonstrated the highest ability against both species. Data demonstrate the mean of three biological replicates. Error bars represent standard deviation (SD). Statistical analysis was performed using one‐way ANOVA to compare the control and treated samples. (* *p* value *<* 0.05, **** *p* value *<* 0.0001).

For *P. aeruginosa* (Figure 6B), the CYC‐loaded hydrogel achieved the highest efficacy, with a reduction of ∼4.38 log compared with the untreated group, followed by SACC (∼3.93 log) and Ace‐K‐loaded hydrogel (∼2.19 log), while the control hydrogel showed a 0.97 log. Given the prevalence of *P. aeruginosa* in chronic wounds and its role in biofilm formation, the antimicrobial properties of AS‐loaded hydrogels present a promising therapeutic approach for managing complex wound infections.

### 3.7 AS Loaded‐Hydrogels Have a Favorable Cytocompatibility Profile

Haemocompatibility refers to a material's ability to come into contact with blood without inducing adverse reactions, which is an important factor for biomaterials. Poor blood compatibility can lead to unwanted physiological responses such as immune responses, thrombosis, and haemolysis [34]. In this study, the blood compatibility of AS‐Loaded PVA hydrogels was studied (Figure 7A). All the hydrogels have an average haemolysis rate below 1.5%, which is significantly lower than the Advancing Standards Transforming Markets (ASTM) F756‐17 standard of 5%. Furthermore, the haemolysis rates of all hydrogels are considered nonhaemolytic according to the ASTM standard (0%–2% = nonhaemolytic, 2%–5% = slightly haemolytic and 5% above = haemolytic). Cell viability is widely used to assess the cytotoxicity and biocompatibility of materials for wound‐dressing applications. To assess the potential cytotoxicity of our AS‐loaded PVA hydrogels, we exposed them to human dermal fibroblasts. Each of the formulations exhibited excellent cell viability compared with the blank control, with no significant differences (Figure 7B). Moreover, all groups treated with hydrogel showed less than 30% reduction and can be considered noncytotoxic [35].

**FIGURE 7:**
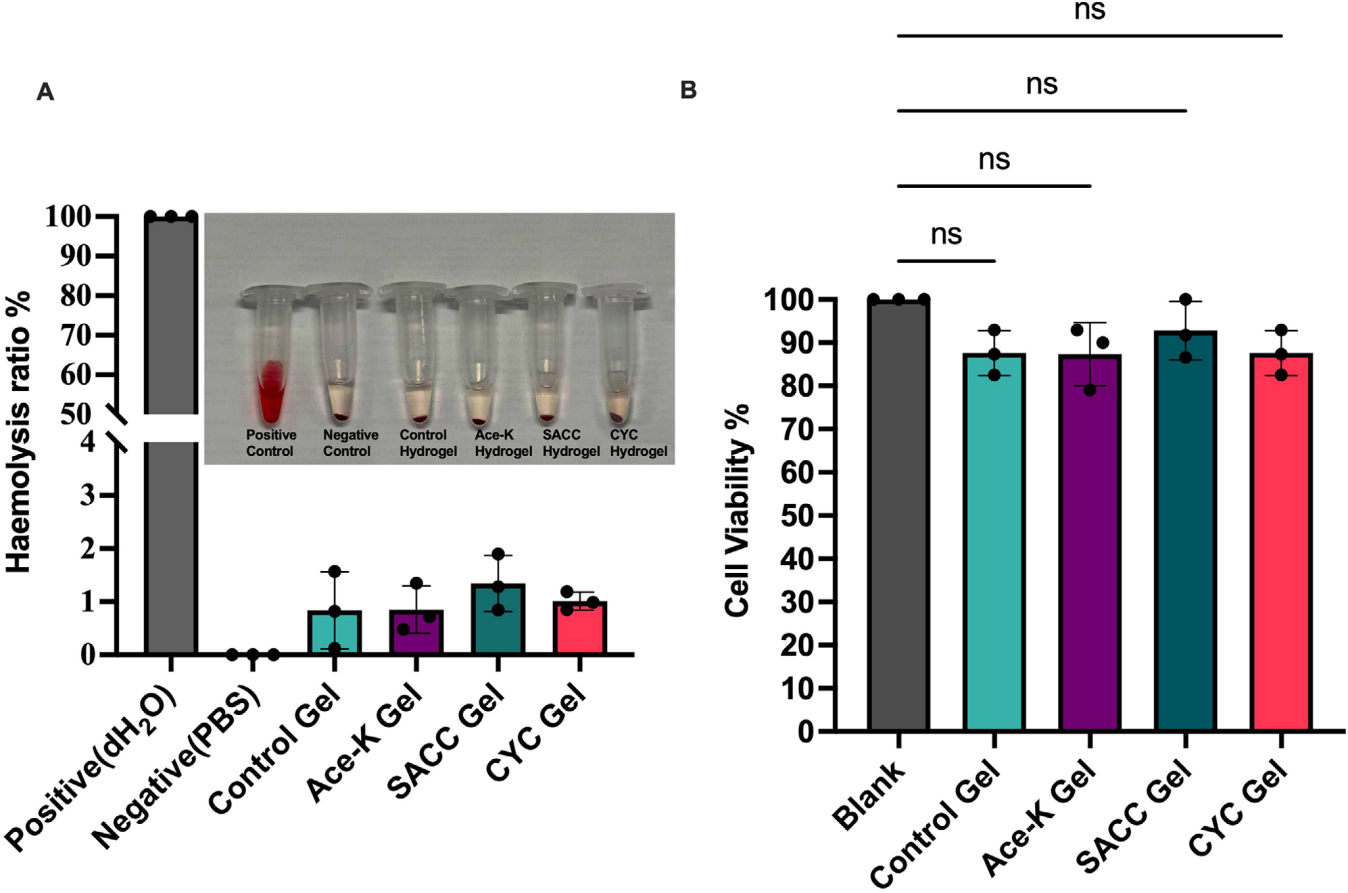
Blood compatibility and cell viability of AS‐Loaded hydrogel. (A) In vitro haemolysis assay of AS‐Loaded hydrogels. (B) Human dermal fibroblast viability percentage. Data are presented as the mean of three biological replicates. Error bars represent standard deviation (SD). Statistical analysis was performed using one‐way ANOVA to compare the control and treated samples. (ns *p* value > 0.05, **** *p* value ≤ 0.0001).

### 3.8 Linking Material Properties to Biological Activity

Having demonstrated the potent antimicrobial activity of the AS‐loaded hydrogels, we next investigated their material properties to gain further insight into their therapeutic potential. To assess the water‐absorption and retention capacity of the hydrogels, the degree of swelling was measured. Swelling behavior is a critical factor for blood and wound exudate, the release of loaded agents and nutrients and metabolites transfer [36]. The degree of swelling (DS) of AS‐loaded PVA‐Borax hydrogel was tested over a 48‐hour period. The control gel had the highest swelling rate compared to the AS‐loaded hydrogels during the study (Figure 8). Maximum average swelling was observed at the third hour (Figure 8B). The control hydrogel reached an average DS of 498.25 ± 18.57%, significantly higher than all other groups. In contrast, the 6% and 8% Ace‐K hydrogels showed lower swelling degrees of 217.51 ± 10.11% and 187.44 ± 85.71%, respectively. Similarly, the 6% and 8% SACC hydrogels exhibited swelling degrees of 212.54 ± 18.25% and 167.93 ± 7.05%, while the 6% and 8% CYC hydrogels showed 219.73 ± 13.71% and 189.67 ± 54.54%, respectively. At 6 h, the control group maintained the highest DS (511.8% ± 58.68), while AS hydrogels exhibited lower swelling values compared with those observed at 3 h. The 6% and 8% Ace‐K hydrogels had swelling degrees of 182.9± 14.82% and 155.4 ± 73.76%, the 6% and 8% SACC hydrogels showed 200.9% ± 23.96 and 136.2 ± 20.14%, and the 6% and 8% CYC hydrogels achieved 187.35 ± 10.99% and 173.57 ± 57.52%, respectively. By 48 h, the control group maintained significantly higher DS (297.3 ± 177.29%) than all other groups. The DS of Ace‐K groups decreased to 147.4 ± 4.94% (6%) and 97.1 ± 38.35% (8%), the SACC groups were 158.6 ± 23.80% (6%) and 110.3 ± 18.03% (8%), and the CYC groups were 154.74 ± 8.27% (6%) and 125.64 ± 36.71% (8%), where a similar trend of less swelling being observed as the AS concentration increases (Figure 8A). This high swelling capacity is due to PVA's hydrophilic surface [37].

**FIGURE 8:**
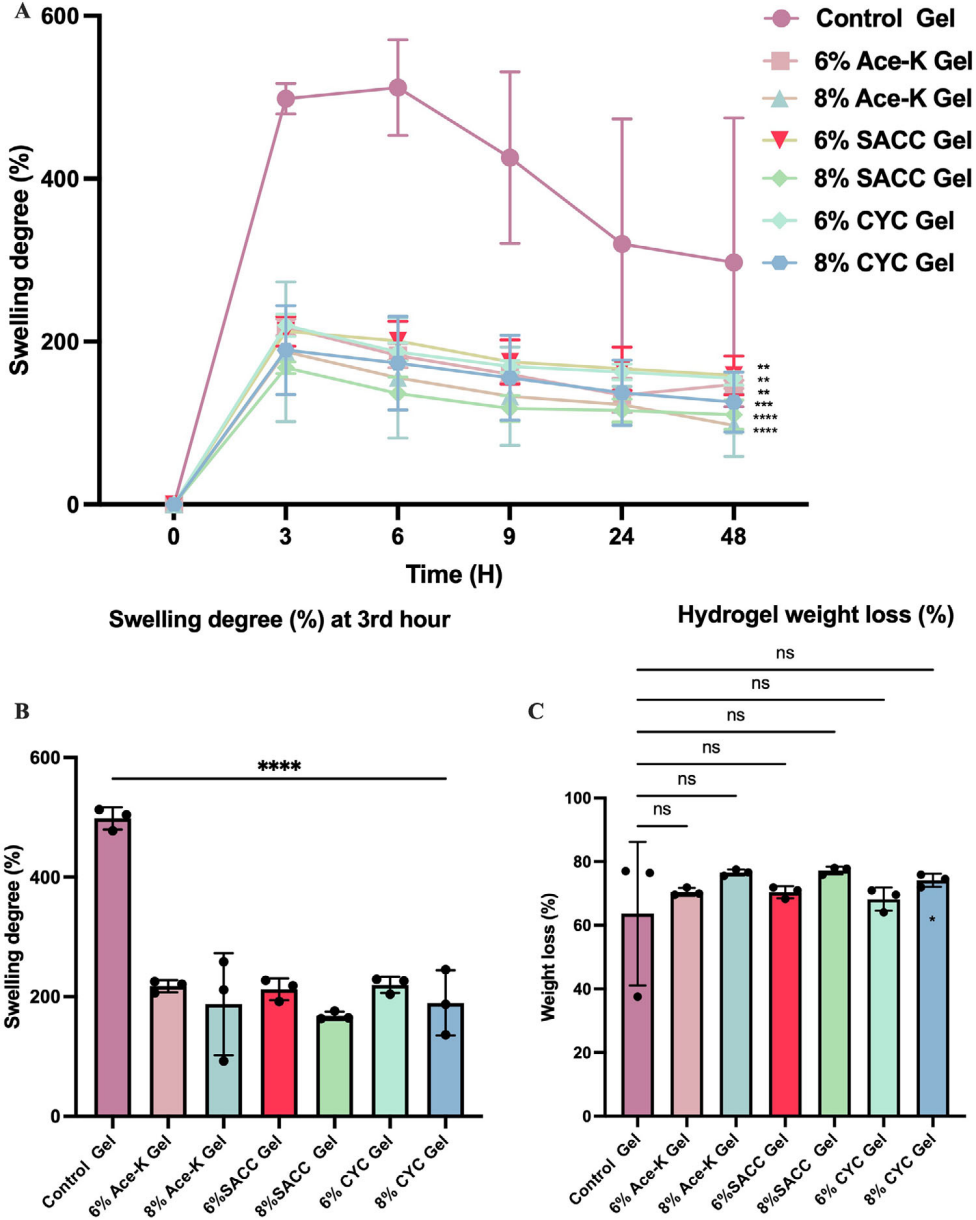
Swelling and WL of hydrogels, (A) Swelling degree (%) of PVA–borax hydrogels incorporated with different AS over a 48‐hour period. Hydrogels were measured at 3, 6, 9, 24, and 48 h. The control hydrogel exhibits the highest SD% among the hydrogels. (B) Comparison of the average swelling degree at 3 h among the control and AS‐loaded groups. The control hydrogel shows the highest DS%. The lower the AS concentration, the higher the DS%. (C) WL (%) was measured at 48 h after re‐dry, the hydrogels containing 6% or 8% (w/w) of Ace‐K, SACC, or CYC, along with a control hydrogel. The WL shows concentration‐dependent behavior compared with AS‐loaded hydrogels. Data are presented as mean ± standard deviation (n = 3). Error bars represent standard deviation (SD). Statistical analysis for A was using two‐way ANOVA with Tukey correction between the AS‐loaded hydrogel and the control hydrogels. Statistical analysis for B, C were performed by one‐way ANOVA with Dunnett's correction between the control and AS‐loaded hydrogel. (Significance for A‐C is indicated as ns *p* > 0.05; *p <0.05; **p < 0.01; ***p < 0.001; ****p < 0.0001).

Hydrogel Weight Loss (WL)is an important parameter for wound dressings. It reflects the hydrogel's stability and degradation profile under physiological conditions [38]. The WL (%) of AS‐loaded PVA‐borax hydrogel was measured (Figure 8C). The control hydrogel exhibited the average WL at 63.66 ± 22.61% and was lower than other hydrogels. Hydrogels loaded with 8% sweeteners generally showed higher WL than the control hydrogel and the 6% AS‐loaded hydrogel. The average WL of 8% Ace‐K hydrogel was 76.57 ± 0.99%, 77.21 ± 1.19% for 8% SACC hydrogel, and 74.12 ±2.01% for 8% CYC hydrogel. Hydrogels containing 6% Ace‐K, 6% SACC, and 6% CYC showed slightly lower WL than 8% hydrogels. The values were 70.49 ± 1.25%, 70.38 ± 1.89%, and 68.19 ± 3.64%.

Although the percentage WL of the AS‐loaded hydrogel was higher than that of the control hydrogel, this does not imply that the AS‐loaded hydrogel degrades more rapidly. As shown in Table 2, their final average weights were similar to those of the control gel. The control gel shows lower WL because the AS is highly water‐soluble.

**TABLE 2:** Initial and final dry weights of AS‐loaded PVA–Borax hydrogels for WL evaluation.

	Controls	6% Ace‐K	8% Ace‐K	6% SACC	8% SACC	6% CYC	8% CYC
Initial dry weight (g)	0.133±0.015	0.262±0.004	0.324±0.017	0.262±0.007	0.3192±0.046	0.259±0.007	0.332+0.031
After dry weight (g)	0.0 46±0.023	0.077±0.0037	0.076±0.0062	0.077±0.0049	0.072±0.0098	0.083±0.0117	0.086±0.0020

The pore structure, size, and porosity can influence swelling rate, fluid retention, and mechanical properties [39]. Different hydrogel pore sizes are better suited to some applications than others. For example, for neovascularization applications, 5 µm is ideal, and 5‐15 µm is more suitable for fibroblast growth. In skin regeneration of adult mammals, 20‐125 µm is the optimal [13]. The hydrogel morphology is shown in Figure 9. The control hydrogel showed a smaller average pore size of 12.00 ± 9.83 µm. The average pore size of Ace‐K hydrogel is 20.75 ± 14.31 µm. The CYC hydrogel shows a slightly larger average pore size (14.38 ± 8.27 µm) when compared to the control hydrogel. The SACC hydrogel showed the largest pore size at 27.42 ± 20.03 µm.

**FIGURE 9:**
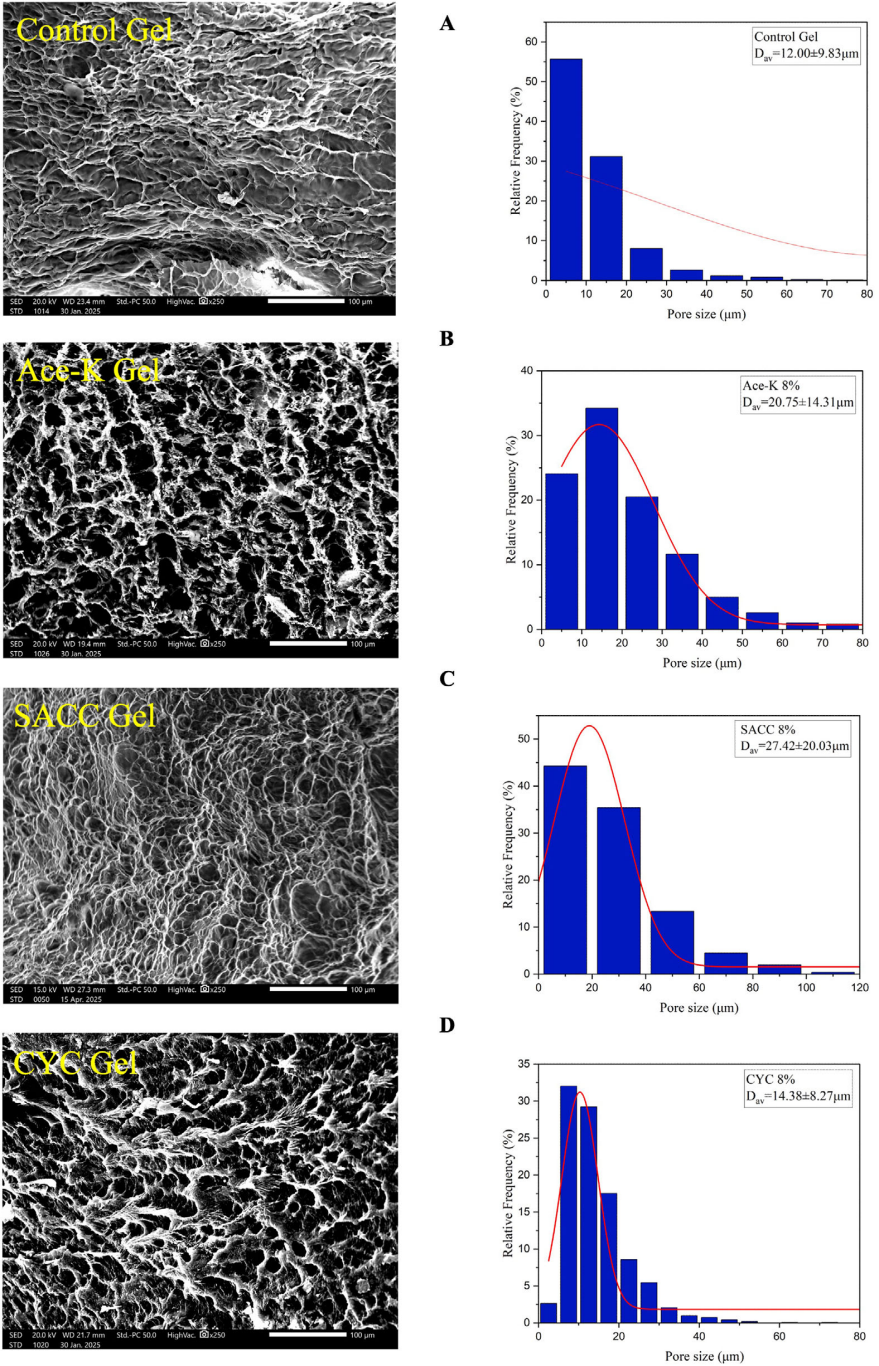
AS‐Loaded Hydrogel Morphologies. Visualization of different pore morphologies of PVA Borax hydrogels with different AS (Control gel, Ace‐K 8%, SACC 8%, and CYC 8%) using SEM. Nonlinear curve fitting on OriginPro software with the Gaussian as the peak function was used for pore size distributions of PVA borax hydrogels.

It is important to understand the rheological behavior of hydrogels, as it provides insight into their mechanical stability. To evaluate the mechanical properties of the AS‐loaded PVA–borax hydrogels, rheological tests were conducted using three formulations containing 8% AS (SACC, CYC and Ace‐K) and a control hydrogel. In Figure 10A, the SACC‐loaded hydrogel has the highest storage modulus and loss modulus in the frequency scanning range of 0‐15.92 Hz, followed by the CYC and Ace‐K‐loaded hydrogel. All AS‐loaded hydrogels exhibited a higher storage modulus compared to the control gel, while their loss modulus remained lower than the storage modulus across the frequency scanning range of 0–15.92 Hz. All the gels showed a higher storage modulus (G′) than the loss modulus (G″), where G′ represents the elastic behavior and G″ represents the viscous behavior. This observation indicates stronger gel intensity [40]. In addition, the higher (G′) value observed in the SACC‐loaded hydrogel indicates a stronger and more rigid network structure compared to other formulations.

**FIGURE 10:**
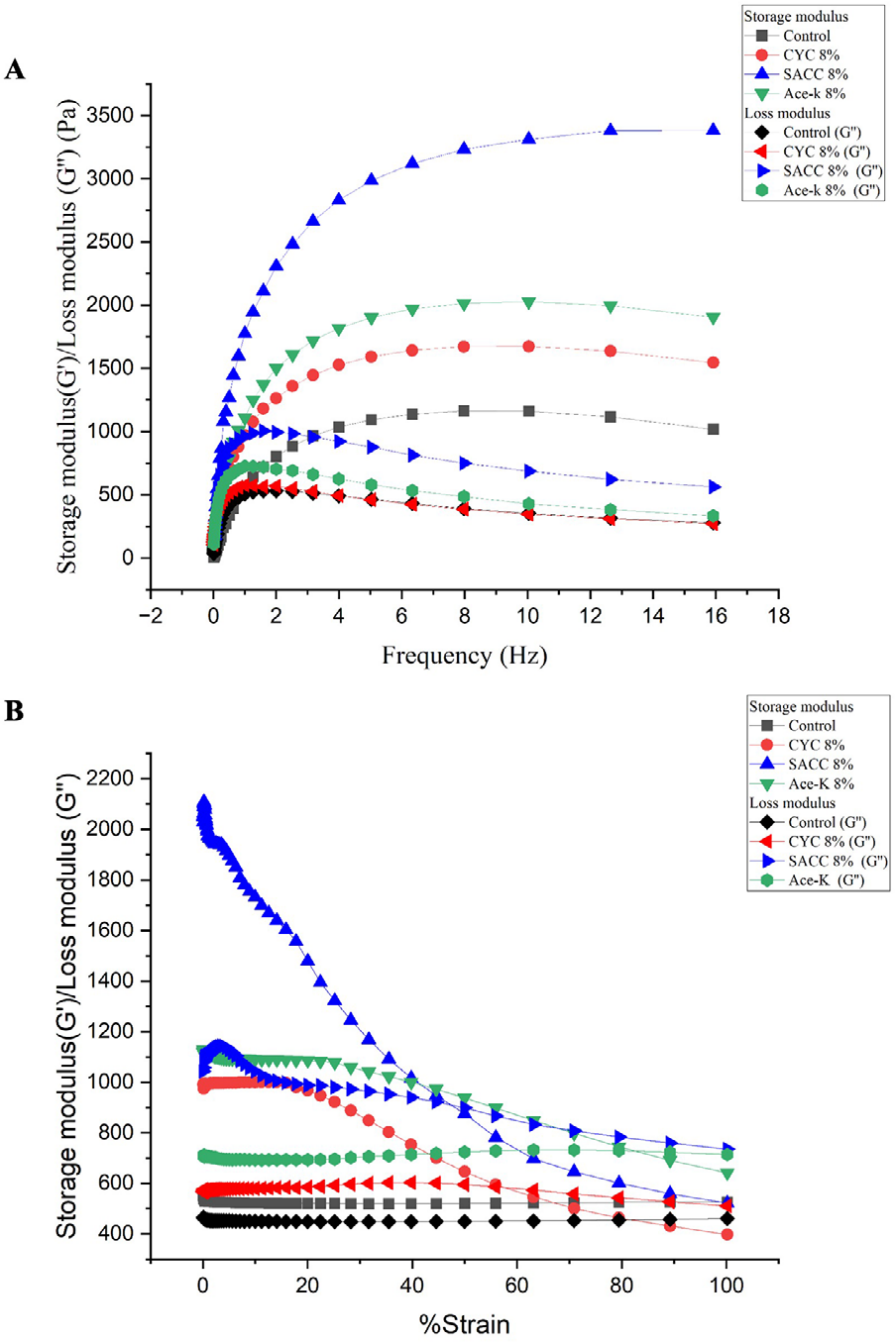
Rheological properties of control and 8% AS‐loaded PVA–borax hydrogels. Rheological measurements were performed using oscillatory rheometry, where the storage modulus (G′) indicates elastic behavior and the loss modulus (G″) indicates viscous behavior. (A) Frequency sweep analysis showing storage modulus (G′) and loss modulus (G″) as a function of frequency (0–15.92 Hz). The SACC‐loaded hydrogel exhibited the highest storage modulus, indicating increased stiffness, while all hydrogels showed G′ values greater than G″ across the frequency range, demonstrating elastic solid‐like behavior. (B) Strain sweep analysis showing storage modulus (G′) and loss modulus (G″) as a function of strain (0%–100%). All hydrogels exhibited decreasing G′ and G″ with increasing strain, indicating shear‐thinning behavior. Data represent mean values from three biological replicates.

Figure 10B shows the variation in storage modulus (G′) and loss modulus (G″) of AS‐loaded hydrogels under increasing strain (%) (0%–100%), enabling quantitative evaluation of their viscoelastic behavior. At lower strains, AS‐loaded hydrogels showed higher G′ than G″, which indicates that the material behaved more solid‐like. With increasing strains, AS‐loaded hydrogels reached a “crossover point” where the G″ started to have higher values than G′, and the gel became more liquid‐like and could flow. The crossover strain values were approximately 82% for Ace‐K, 47.5% for SACC, and 58.5% for CYC while control sample showed no clear crossover within 0‐100 strain%. This shows the AS‐loaded gels are non‐Newtonian shear‐thinning fluids. The SACC‐loaded PVA borax demonstrated the highest storage modulus across the entire strain range. The Ace‐K‐loaded hydrogel showed a moderately high storage modulus, slightly lower than SACC but higher than CYC and control hydrogels. The CYC‐loaded hydrogel exhibited a storage modulus similar to or slightly higher than the control hydrogel. This enhanced rigidity can improve the hydrogel's stability on the wound surface, which complies with the pilot study.

Fourier Transform Infrared Spectroscopy (FTIR) analysis was performed to identify the functional groups and molecular interactions in the PVA–borax hydrogels. It is also used to confirm if there is any chemical interaction between AS and PVA–borax hydrogels. In the FTIR spectrum of PVA, the characteristic peak at approximately 3250 cm^−1^ is assigned to the O─H stretching vibration of hydroxyl groups. The characteristic peaks at 2940 cm^−1^ and 2905 cm^−^^1^ are attributed to CH_2_ asymmetric stretching vibrations. The peak at 1415 cm^−^^1^ corresponds to the C─H bending vibration of ─CH_2_‐ groups, while the peak at 1320 cm^−^^1^ represents the CH and OH bending groups. The absorption peak at 1145 cm^−^^1^ indicates the C─O stretching vibration of the ─CH_2_OH hydroxyl groups in PVA molecules, which was previously described (Figure 11) [41, 42, 43]. In Figure 11, the PVA hydrogel shows a broad peak at 3250 cm^−^^1^, which indicates stretching vibration of the O─H group. Peaks at 2940 cm^−^^1^ and 2905 cm^−^^1^ were assigned to C─H stretching, and bands at 1415 cm^−^^1^ and 1320 cm^−^^1^ corresponded to ─CH_2_ bending and CH+OH bending groups. A reduced peak at 3250 cm^−^^1^ means the number of hydroxyl species reduced. This reduction indicated the formation of a boric ester bond [44]. In Ace‐K‐loaded PVA hydrogels (Figure 11A), characteristic peaks of Ace‐K were observed at 1650 cm^−^^1^ (C═O stretching), 1590 cm^−^^1^ (N─H bending), and 1075 cm^−^^1^ (S═O stretching), confirming that there is no chemical interaction of Ace‐K with the hydrogel matrix [45]. The B─O stretching vibration at 997 cm^−^^1^ remained visible in the Ace‐K‐loaded PVA hydrogels, indicating the presence of borate crosslinking. In Figure 11B, CYC‐loaded hydrogels showed a sharp N─H stretching peak at 3275 cm^−^^1^ and a broad O─H stretching band (3200–3500 cm^−^^1^). Other peaks were found to be at 3275 cm^−^^1^, 2935 cm^−^^1^ and 2856 cm^−^^1^ (C─H stretching), 1432 cm^−^^1^ (C─H bending); 1170 cm^−^^1^ and 1035 cm^−^^1^ (S═O stretching); and 683 cm^−^^1^ (C─S bending), which substantiate the addition of CYC in the hydrogel system [46]. In Figure 11C, SACC‐loaded hydrogels showed clear peaks at 3567 cm^−^^1^ and 3487 cm^−^^1^ corresponding to O─H stretching and hydrogen bonding, which match the SACC powder spectra. Aromatic stretching C═C (1630 cm^−^^1^ and 1583 cm^−^^1^), the sulfonyl group peak (1255 cm^−^^1^ and 957), and C─S‐related peaks at 748 cm^−^^1^ and 674 cm^−^^1^ were identified both in SACC powder and SACC‐loaded hydrogel [47]. The FTIR spectra confirm the successful incorporation of AS into the PVA‐borax hydrogel network, and there was no chemical interaction between the PVA‐borax hydrogel and AS.

**FIGURE 11:**
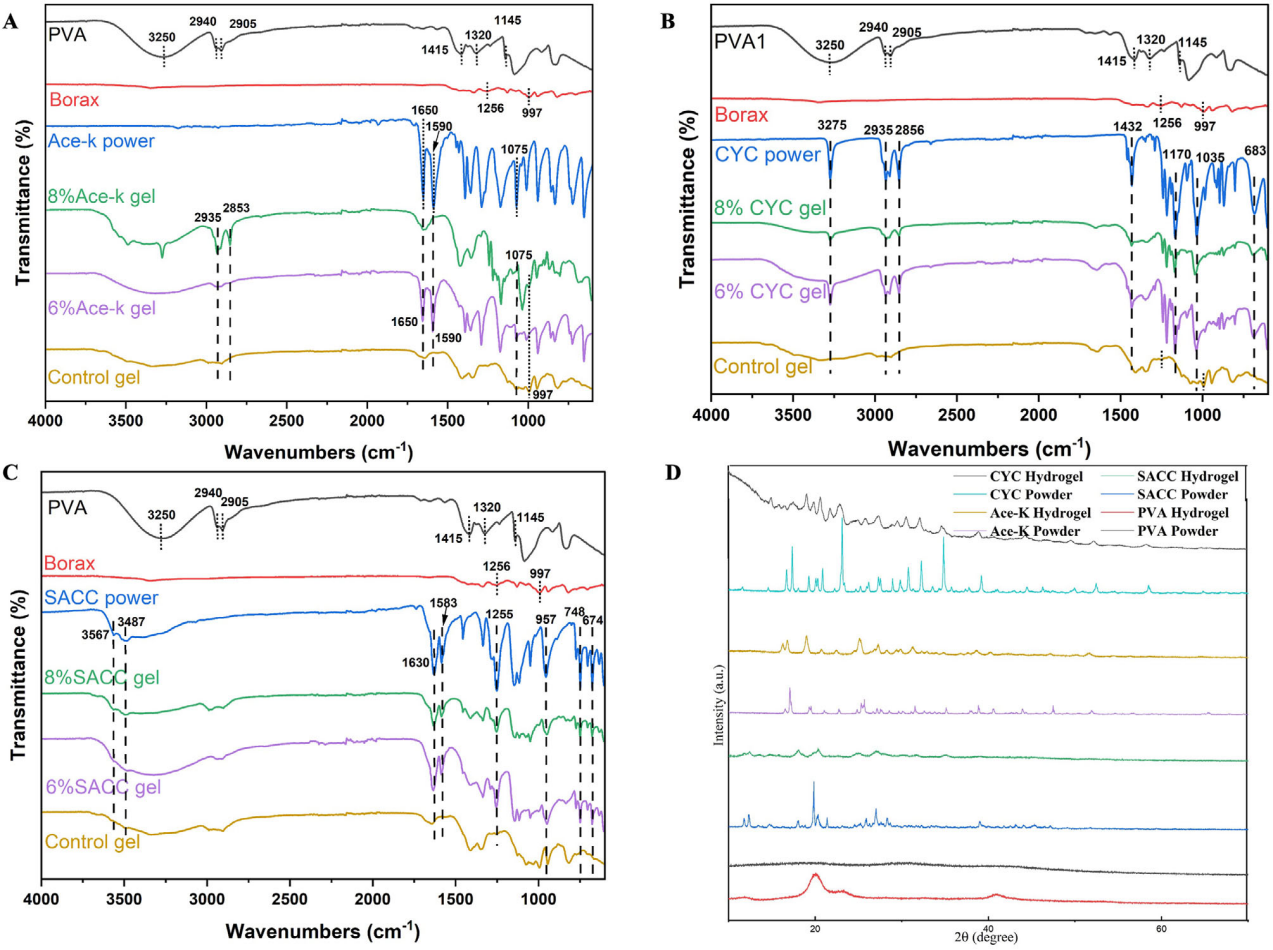
FTIR, XRD Analysis of AS‐Loaded Hydrogels. (A) FTIR spectra of PVA, sodium tetraborate, Ace‐K, 8% Ace‐K hydrogel, 6% Ace‐K hydrogel, and control hydrogel. (B) Sodium tetraborate, CYC, 8% CYC hydrogel, 6% CYC hydrogel and control hydrogel. (C) FT‐IR spectra of PVA, sodium tetraborate, SACC, 8% SACC hydrogel, 6% SACC hydrogel, and control hydrogel. (D) XRD spectra of PVA powder, PVA hydrogel, CYC powder, CYC Hydrogel, SACC powder, SACC hydrogel, Ace‐K powder, Ace‐K hydrogel.

The x‐ray diffractometry (XRD) patterns of AS‐Loaded hydrogels are presented in Figure 11D. The PVA powder exhibited a sharp diffraction peak at 2θ = 20.03°, indicating its semi‐crystalline nature. This crystallinity arises from strong intermolecular hydrogen bonding between PVA chains, as previously reported [48]. In contrast, the PVA‐borax hydrogel exhibited two broad peaks (Figure S3), suggesting a significant reduction in crystallinity. This change is attributed to borax crosslinking, which sterically interferes with the regular arrangement and hydrogen bonding of PVA chains within the network structure [44]. The AS samples exhibited characteristic crystalline peaks, confirming their crystalline structure (Figure 11D). After incorporation into the PVA hydrogel matrix, the broad diffraction peak of the PVA hydrogel disappeared and was replaced by the characteristic peaks of AS. However, the intensity of these AS peaks decreased compared to pure AS, indicating reduced crystallinity. This reduction suggests that the AS are molecularly dispersed or partially embedded within the amorphous regions of the hydrogel network, thereby distorting its ordered crystalline structure.

### 3.9 Pilot Phase 1 Clinical Study in Humans

To evaluate whether the AS hydrogel causes any effects on healthy human skin, such as irritation, discomfort, or other adverse events, a pilot Phase I clinical study was conducted. In this study, 12 healthy volunteers were recruited, and all of them completed the study. The 4 different hydrogels (Control, 8% Ace‐K, 8% SACC and 8% CYC) were placed on their healthy, unbroken skin. To keep the hydrogel in place, adhesive film was used (Figure 12A). This study also aimed to assess potential issues related to the adhesion and removal of the hydrogel from the skin, as well as its degradation over time. The pH of the hydrogels was measured before the pilot study, and the average was 8.07 (Figure S4). In the Participant Feedback on Hydrogel Application Study—Section 1: Comfort and Sensation, the majority of participants (75%) rated the hydrogel as highly comfortable (score of 5). The remaining 25% rated it as 4, suggesting it was well‐tolerated (Figure 12B). One participant reported an increasing comfort sensation over time (Figure 12C). After 24 h, 50% of participants rated the sensation as 5, and 25% rated it as 4 (Figure 12D). The majority of participants did not experience discomfort, but one participant reported itchiness due to the adhesive tape securing the hydrogel [49] (Figure 12E).

**FIGURE 12:**
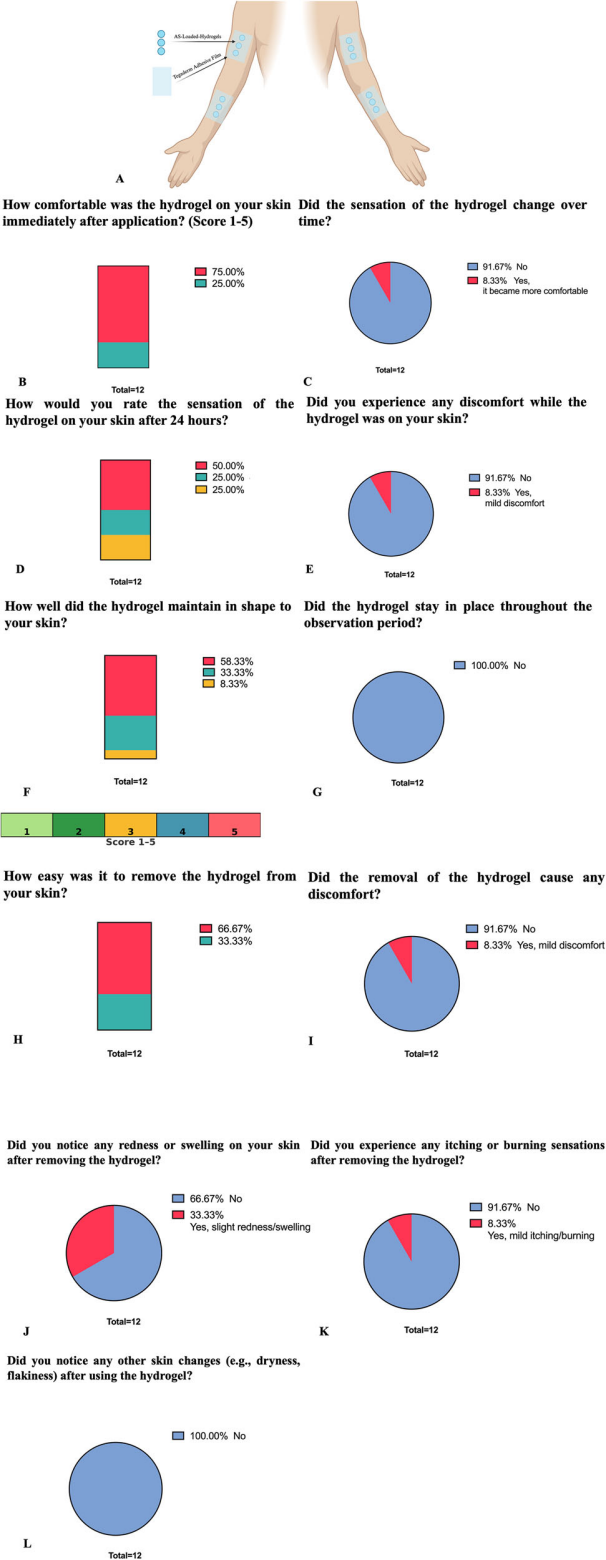
Pilot Phase 1 Trial: (A) illustration of how hydrogels were fixed by Tegaderm adhesive film (3 M) to mimic clinical dressing conditions for 24 h. (B‐E) Feedback from 12 Participants on hydrogel application study—Section 1: Comfort and sensation. (F‐I) Section 2: Adhesion and (J‐L) Removal Section 3: Skin Reaction Created in BioRender. Han, J. (2025) https://BioRender.com/6sdwcby.

In the adhesion and removal section, the feedback shows that 58.33% of participants rated the hydrogel's stability as excellent, with 33.33% rating it as 4 and 8.33% giving it 3 for structural integrity (Figure 12F). All participants reported that phase separation occurred in both the CYC and Ace‐K hydrogels during the test. Most participants experienced 8% CYC hydrogel phase separation within the first 3 h. However, there were two participants (both male) whose CYC hydrogels phase separated at the sixth‐hour observation point. This could be due to males typically having higher skin temperatures than female [49, 50]. Ace‐K‐loaded hydrogel phase separated between the 9‐ and 24‐hour time points. Hydrogels loaded with SACC and the control hydrogels maintained their structural integrity over 24 h (Figure 12G). The removal processing of the hydrogels was relatively easy. There were 66.67% of participants who found the process very easy (score of 5), and 33.33% found it slightly difficult (score of 4) (Figure 12H). One participant reported discomfort due to the strong adhesive of the transparent film (Figure 12I).

In the skin reaction section, four participants experienced redness or swelling post‐hydrogel removal. These reactions were localized to the area where the transparent film was applied, rather than the hydrogel application site (Figure 12J). This is due to repeated application and removal of the adhesive film, which irritated the skin. Moreover, one participant reported an itching/burning sensation that was also confined to the area covered with the adhesive film (Figure 12K). None of the participants observed any skin changes, such as dryness or flakiness, after hydrogel application (Figure 12L). These findings suggest that while a minority of participants experienced mild and transient redness and itching due to the commercially available transparent film that was on top of the hydrogels, the majority reported no adverse effects. The reactions were limited to the site of the transparent film and not on the AS‐loaded hydrogel sites, indicating that the AS‐loaded hydrogels are well tolerated on healthy skin during the applications.

## 4 Discussion

The global rise of AMR has created an urgent need for novel antimicrobial agents. The exploration of unconventional compounds that have antimicrobial activity provides a new avenue as traditional antibiotics become less effective against multidrug‐resistant pathogens. Moreover, the timeframe from lab bench to clinical use of a new antimicrobial is 10‐15 years, and it could cost US$1.4 billion per antibiotic [51]. Repurposing known compounds with antimicrobial activity to treat infection could save US$1 billion and half the time required for clinical translation [52].

AS are ubiquitous in our lives. The market scale of AS is increasing every year [53, 54]. In our previous studies, AS such as Ace‐K, SACC and CYC demonstrated the ability to inhibit the growth of common wound infection AMR pathogens, including the *A. baumannii* (AB5075) and *P. aeruginosa* (PA14) [10,20]. We also demonstrated a range of antivirulence properties including the inhibition of biofilm formation, natural transformation and motility. In this prior work, we extensively explored the underlying mechanism of action using bacterial cytological profiling and transcriptomics, uncovering that these AS triggered the downregulation of key genes associated with these phenotypes and that cell death was mediated via bulge mediated cell lysis. This study focused on the translational potential of these sweeteners by developing novel formulations for their topical application. Specifically, we developed a PVA‐borax hydrogel platform and assessed its suitability as a delivery system for Ace‐K, SACC and CYC. An initial screening using the ZOI assay was conducted to assess the antimicrobial potential of hydrogels loaded with different AS concentrations. Interestingly, the AS‐loaded hydrogels showed a concentration‐dependent effect consistent with our previous findings, which used AS solutions directly ([10], 2025). This demonstrated the efficacy of this hydrogel as a vehicle for the AS and that they maintained their activity. As 8% AS‐loaded hydrogels showed the highest ZOI, they were selected for testing against biofilm.

Biofilm is a leading factor that contributes to prolonged wound healing [55, 56]. It leads to a burden of $386.8 billion on health systems globally [57]. The antibiofilm activity of our AS‐loaded hydrogels was tested against early‐stage biofilm (3.5 h) and mature biofilm (24 h). For early‐stage biofilm, an in vitro and ex vivo porcine skin model was evaluated. There was a ≥ 99.99% reduction in viable biofilm cells observed after 1‐hour treatment in the in vitro model. This was also confirmed in the ex vivo porcine skin model, where all AS‐loaded hydrogels demonstrated a ∼99% reduction in viable cells within the biofilm, whereas the control gel showed no significant efficacy against the biofilm. Moreover, AS‐PVA hydrogels were compared with a commercial silver dressing that serves as a clinically relevant comparator. As a result, all AS‐PVA hydrogels outperformed the commercial silver dressing, which showed no efficacy against early biofilm, highlighting the inefficacy of current frontline therapeutic interventions in treating *A. baumannii* AB5075 and *P. aeruginosa* PA14 biofilm‐associated infections. This confirms the potential use of our dressing in early‐stage wound infections. In the established biofilm model, reduction in viable biofilm cells was also observed within 2 h and increased over time, achieving ≥99.99% biofilm removal in 24 h. SEM analysis confirmed these findings. Interestingly, it was noted that the few remaining cells in the sweetener‐treated groups displayed an altered cell morphology with cells being rounder than their untreated counterparts. This aligns with our previous reporting of the altered *A. baumannii* cell morphology induced by sweetener exposure [10]. We next hypothesized that the hydrogel would allow a controlled release of the AS for continuous eradication of biofilms in chronic wounds. This was further validated by time‐kill assays, which showed that hydrogels loaded with 6% and 8% AS significantly reduced *A. baumannii* viability in PBS over 24 h, and that AS and borax were the only factors affecting cell counts. However, the control hydrogel demonstrated limited inhibition, further validating that loading AS enhances the antimicrobial activity. Future studies will focus on evaluating time‐release kinetics of AS‐loaded PVA hydrogel in host‐relevant media using HPLC analysis. To explore potential real‐world applications, we further demonstrated the antibiofilm activity of the AS‐loaded hydrogels in a dual‐species biofilm model. The *A. baumannii* AB5075 and *P. aeruginosa* PA14 were selected due to their common association with chronic wound infections [58, 59]. Moreover, the AS‐loaded PVA hydrogel demonstrates excellent hemocompatibility, with a haemolytic rate below 2% and excellent cell viability within a noncytotoxic range (less than 30% reduction) [35]. These results demonstrate that AS‐Loaded PVA borate hydrogels have excellent biocompatibility and antimicrobial efficacy. Future studies should investigate the effects of AS‐loaded hydrogels on wound healing and provide more details on fibroblast morphology, including their influence on fibroblast morphology by using fluorescence images of live/dead stain, cell migration, tissue regeneration, and wound closure.

In addition to their antimicrobial efficacy and biocompatibility, the physical properties of AS‐loaded hydrogels were evaluated to understand how material characteristics influence hydrogel performance. The degree of swelling of PVA‐hydrogel was monitored over 48 h. The control gel reached its maximum swelling at 3 h, and that was maintained till 6 h before starting to degrade. In contrast, sweetener‐containing hydrogels showed less swelling compared to the control gel and started to degrade earlier at 3 h. These findings suggest that AS incorporation reduces the swelling capacity of the hydrogel. However, all the hydrogels reached more than 150% swelling at the 3‐hour time point, and a high swelling rate could allow the hydrogel to have a high wound exudate absorption capacity and high drug release, which is ideal for deployment in wound applications [36]. Over time, all groups showed a gradual reduction in swelling, indicating the biodegradability of the hydrogels. The average pore size was around 12.00 ± 9.83 µm for the control gel, 20.75 ± 14.31 µm for Ace‐K gel, 14.38 ± 8.27 µm for CYC gel and 27.42 ± 20.03 µm for CYC gel. However, the swelling rate trend does not align with the porosity that was observed by SEM. These different observations could be due to the high‐water solubility of the AS. The AS hydrogel has a higher initial weight compared to the control hydrogel, and AS are highly water‐soluble. Therefore, AS might rapidly dissolve after liquid immersion, which results in a lower swelling rate.

In this study, the incorporation of AS changed the viscoelastic behavior of the PVA–borax hydrogels. When the storage modulus (G′) is lower than the loss modulus (G″), the material behaves as a viscous fluid. When storage modulus (G′) is higher than the loss modulus (G″), the material behaves as a gel. In the results, it showed that at lower frequencies, the hydrogels tended to exhibit viscous behavior, whereas at higher frequencies, the storage modulus (G′) exceeded the loss modulus (G″), indicating elastic solid‐like behavior under these conditions. Incorporation of AS increased the storage modulus compared to the control hydrogel. Specifically, the storage modulus of the SACC‐loaded PVA–borax hydrogel was approximately three times higher than that of the control gel, while CYC‐ and Ace‐K‐loaded hydrogels exhibited increases of approximately 1.5‐fold and 1.8‐fold.

Given that the AS‐loaded hydrogels had displayed potent antimicrobial and antibiofilm activity, we would like to further explore their therapeutic potential. A pilot Phase I clinical study was conducted to evaluate the subjective experience and skin compatibility of AS‐loaded hydrogels in healthy human participants. The goal was to assess short‐term safety, irritancy and subjective comfort and skin compatibility under conditions simulating topical application. The majority of participants reported that AS hydrogels were comfortable to wear. However, a few cases of mild itchiness and redness were reported after removing the adhesive film. Importantly, these reactions were localized to the adhesive area rather than the hydrogel contact zone, which indicated the hydrogel did not induce irritation. However, repeated application and removal of the adhesive film might cause skin damage in sensitive individuals. To minimize such reactions in future experimental designs, we would reduce the number of application intervals to minimize skin damage.

In terms of formulation stability during wear, hydrogel stability varied among the formulations. SACC hydrogel maintained its integrity during the 24 h application period. In contrast, the CYC hydrogel phase separated within 3 h of application, whereas the Ace‐K hydrogel phase separated between 9 and 24 h. These differences highlight the impact of sweetener type on the mechanical properties of the hydrogel under physiological conditions. The findings reinforce the need for hydrogel formulations to balance not only antimicrobial efficacy but also practical usability and wearability in real‐world applications. Future work will look to conduct a full Phase 1 clinical trial, which will evaluate the effects of dose escalation, pharmacokinetics, with predefined clinical endpoints.

## 5 Conclusion

In conclusion, this study demonstrates that AS‐loaded PVA–borax hydrogels exhibit both antimicrobial and antibiofilm efficacy and favorable material properties for topical application in acute and chronic wounds, compared with the control hydrogel. The hydrogels effectively inhibited *A. baumannii* both in planktonic and biofilm conditions. The antimicrobial activity was observed in both mono‐ and dual‐species models. The swelling and porosity of the hydrogels were influenced by sweetener type and concentration, with solubility playing a key role in observed variability. SEM analysis and adjusted swelling degree calculations further clarified these effects. Importantly, human testing indicated good comfort and biocompatibility, although improvements in adhesive application and hydrogel stability, particularly for Ace‐K and CYC formulations, are essential. Overall, these findings highlight the promise of AS‐loaded hydrogels as an innovative platform for wound care and localized infection management.

## Funding

R.R.M. is supported by a Biotechnology and Biological Sciences Research Council Awards (BB/V007823/1, UKRI) and a Medical Research Council Grant (MR/Y001354/1). R.R.M. is also supported by the Academy of Medical Sciences/the Wellcome Trust/the Government Department of Business, Energy and Industrial Strategy/the British Heart Foundation/Diabetes UK Springboard Award (SBF006∖1040).

## Conflicts of Interest

Brunel University of London and Ronan McCarthy have priority patents covering the therapeutic application of artificial sweeteners.

## Supporting information




**Supporting File**: adhm71618‐sup‐0001‐SuppMat.pdf.

## Data Availability

The data that support the findings of this study are available from the corresponding author upon reasonable request.
