# Supplementary material for: Hydrogels Augmented With Artificial Sweeteners can Inhibit Multidrug‐Resistant Acinetobacter baumannii Growth and Biofilm Formation While Demonstrating Safety in Pre‐Clinical Pilot Human Trials
Source: Adv Healthc Mater. 2026 Aug 26;15(39):e05777. doi: 10.1002/adhm.202505777 (PMC13691406; doi:10.1002/adhm.202505777)
Supplement: Supplementary file 1 [file ADHM-15-0-s001.pdf]

**Supporting Information**

# Hydrogels Augmented With Artificial Sweeteners Can Inhibit Antibiotic-Resistant Pathogen *Acinetobacter baumannii* Growth and Biofilm Formation while demonstrating safety in Pre-Clinical Pilot Human Trials

**Jie Han[1], Mohamed Soliman[1,3], Bin Zhang[2], Dominika Krawiel[1,3], Ronan R McCarthy[1,3]**

[1] Division of Biosciences, Department of Life Sciences, Antimicrobial Innovations Centre, College of Health and Life Sciences, Brunel University London, Uxbridge, UB8 3PH, United Kingdom

[2] Mechanical and Aerospace Engineering Department, Brunel University London, UB8 3PH, United Kingdom

[3] National Biofilms Innovation Centre, School of Biological Sciences, Faculty of Environmental and Life Sciences, University of Southampton, Southampton, S017 1BJ

**Corresponding Author:** ronan.mccarthy@brunel.ac.uk; ronan.mccarthy@soton.ac.uk

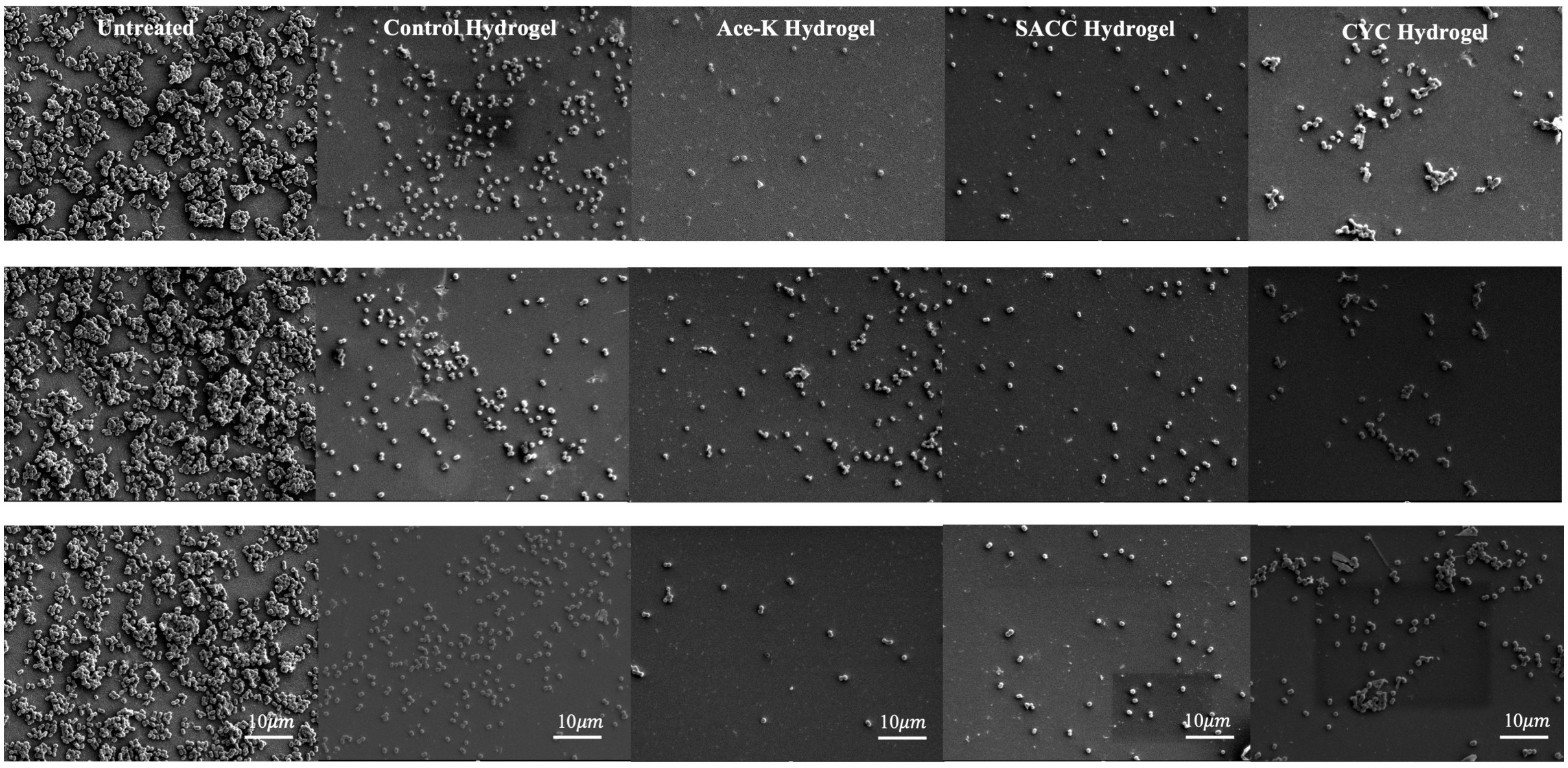


**Figure S1 Scanning electron microscopy (SEM) images showing the effect of AS-loaded PVA hydrogels on *A. baumannii* AB5075 biofilm formation after 24 h incubation.** Untreated samples exhibited dense biofilm coverage and bacterial aggregation on the glass surface. The control hydrogel reduced biofilm density slightly, while Ace-K-, SACC-, and CYC-loaded hydrogels markedly decreased bacterial attachment and biofilm formation.

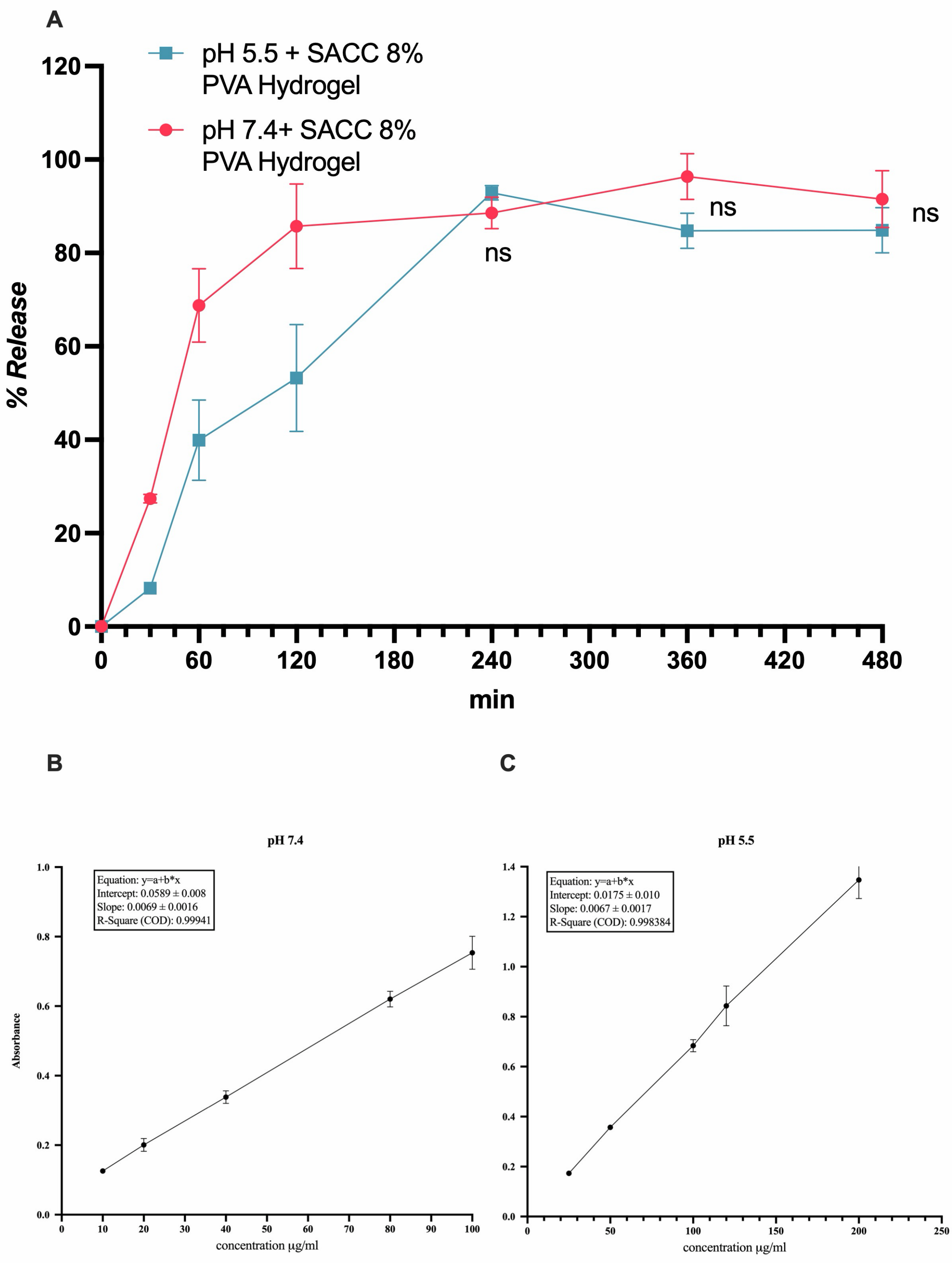
A
pH 5.5 + SACC 8% PVA Hydrogel
pH 7.4+ SACC 8% PVA Hydrogel
% Release
min
ns
ns
ns
B
pH 7.4
Equation: y=a+b*x
Intercept: 0.0589 ± 0.008
Slope: 0.0069 ± 0.0016
R-Square (COD): 0.99941
Absorbance
concentration µg/ml
C
pH 5.5
Equation: y=a+b*x
Intercept: 0.0175 ± 0.010
Slope: 0.0067 ± 0.0017
R-Square (COD): 0.998384
concentration µg/ml

D

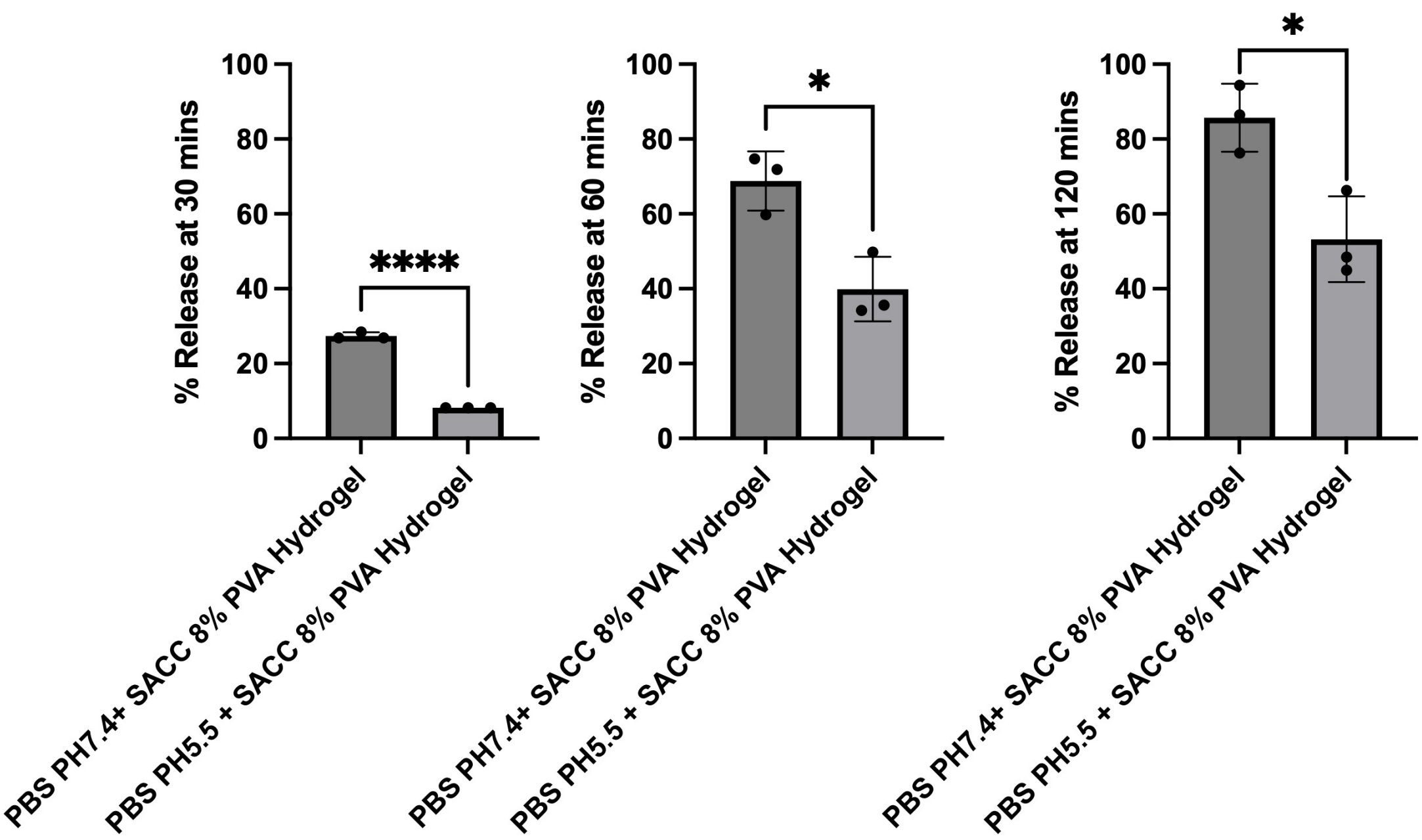


**Figure S2. The absorbance of SACC released from hydrogels under pH 7.4 or pH 5.5 were collected at predetermined time points, 0, 30, 60, 120, 240, 360 and 480 min, aliquots of the release medium were collected and replaced with fresh medium. (A) Release Profiles of SACC from PVA-borate hydrogels (B–C) Standard calibration curves of SACC at pH 7.4 and pH 5.5. (D) Significantly different time points of SACC Release from 8% SACC PVA hydrogels under different pH conditions.** Cumulative release (%) of SACC over 480min from 8% SACC-Loaded PVA borate hydrogel. The 8% SACC-loaded PVA borate hydrogel showed faster early-stage release at pH 7.4, while at pH 5.5 it exhibited more controlled release behaviour at later time points. Data are presented as mean ± SD (n = 3). (ns>0.05, * p value < 0.05, **** p value < 0.0001).

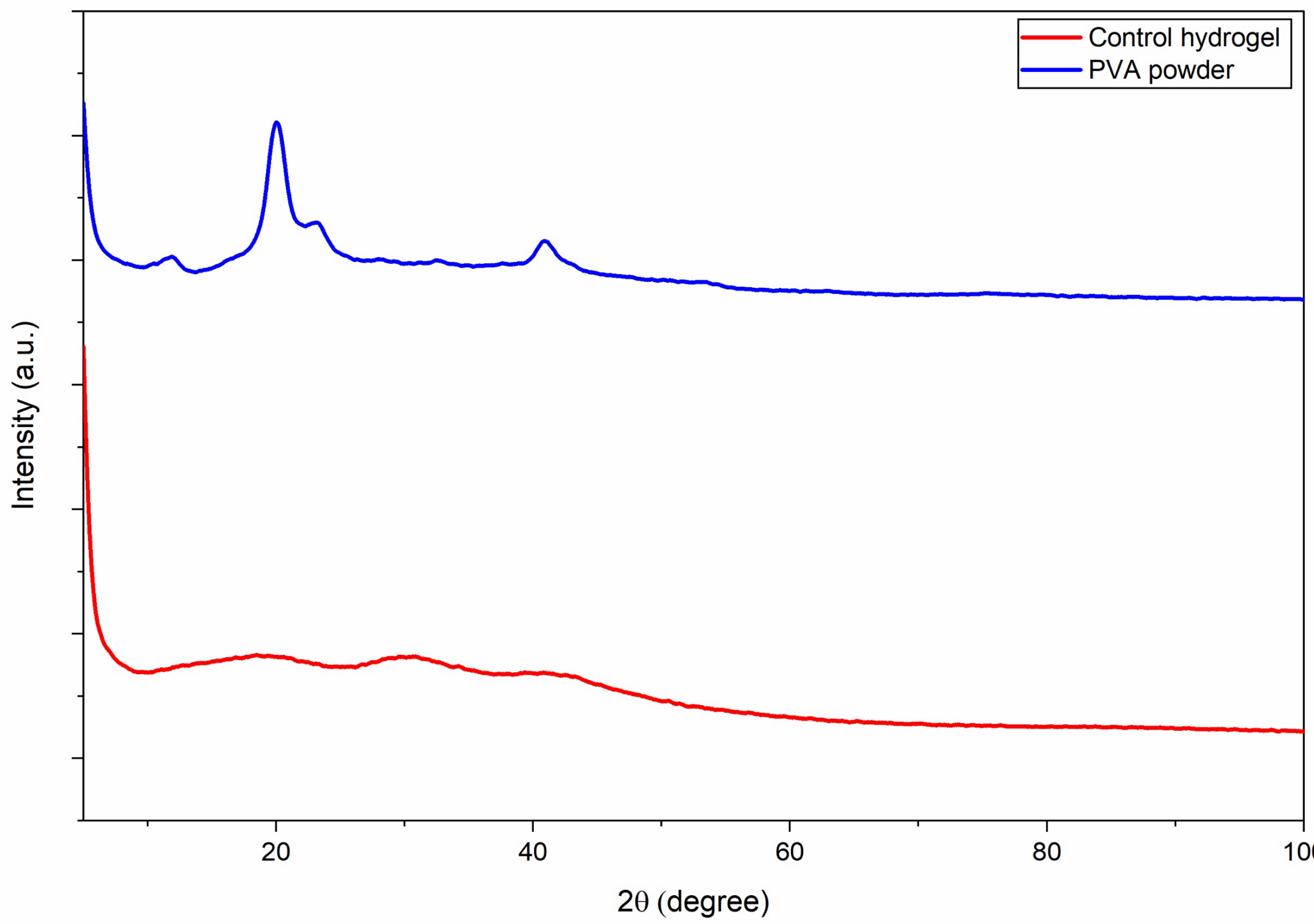


**Figure S3 XRD Spectra of the PVA powder and PVA-Hydrogel**

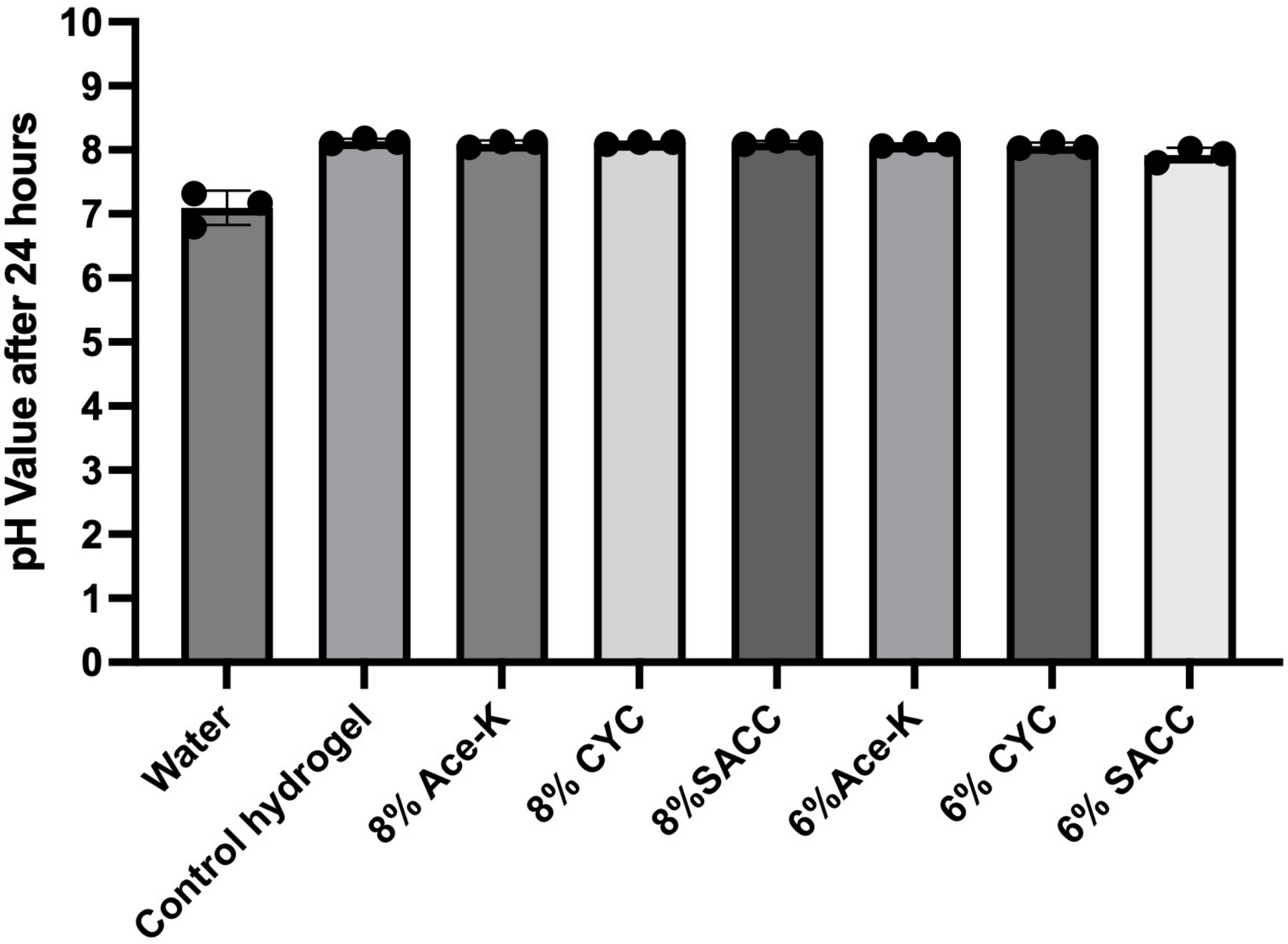


**Figure S4 pH Value of AS-loaded PVA-Hydrogels.** Hydrogels were immersed in water for 24 hours before pH measurement. (n=3)

College of Health, Medicine and Life Sciences Research Ethics Committee (DLS)
Brunel University of London
Kingston Lane
Uxbridge
UB8 3PH
United Kingdom

www.brunel.ac.uk

21 November 2024

**CONDITIONAL LETTER OF APPROVAL**

APPROVAL HAS BEEN GRANTED FOR THIS STUDY TO BE CARRIED OUT BETWEEN 21/11/2024 AND 30/09/2025

Applicant (s): Miss Jie Han

Project Title: Testing Artificial Sweetener-Loaded PVA Hydrogel's Effect on Healthy Human

Reference: 47765-MHR-Nov/2024- 53276-3

Dear Miss Jie Han

The Research Ethics Committee has considered the above application recently submitted by you.

The Chair, acting under delegated authority has agreed that there is no objection on ethical grounds to the proposed study. Approval is given on the understanding that the conditions of approval set out below are followed:

**PIS - Voluntary Participation and Withdrawal – please remove "you may only withdraw your data up to two weeks after the study. After this point, the data will have been anonymized and cannot be linked back to you for removal." You have already confirmed that participants can withdraw their data by 30th September 2025. The former sentence is contradictory.**

**Please update the PIS and send in via correspondence for checking before commencing the study. Please also ensure you update any relevant documents with the ethical approval start date.**

- **The agreed protocol must be followed. Any changes to the protocol will require prior approval from the Committee by way of an application for an amendment.**

Please note that:

- Research Participant Information Sheets and (where relevant) flyers, posters, and consent forms should include a clear statement that research ethics approval has been obtained from the relevant Research Ethics Committee.
- The Research Participant Information Sheets should include a clear statement that queries should be directed, in the first instance, to the Supervisor (where relevant), or the researcher. Complaints, on the other hand, should be directed, in the first instance, to the Chair of the relevant Research Ethics Committee.
- Approval to proceed with the study is granted subject to any conditions that may appear above.
- The Research Ethics Committee reserves the right to sample and review documentation, including raw data, relevant to the study.
- If your project has been approved to run for a duration longer than 12 months, you will be required to submit an annual progress report to the Research Ethics Committee. You will be contacted about submission of this report before it becomes due.
- You may not undertake any research activity if you are not a registered student of Brunel University or if you cease to become registered, including abeyance or temporary withdrawal. As a deregistered student you would not be insured to undertake research activity. Research activity includes the recruitment of participants, undertaking consent procedures and collection of data. Breach of this requirement constitutes research misconduct and is a disciplinary offence.

Professor Louise Mansfield

Chair of the College of Health, Medicine and Life Sciences Research Ethics Committee (DLS)

Brunel University London



**Figure 5S. Ethics letter of Approval.**

49 Spadina Ave. Suite 200
Toronto ON M5V 2J1 Canada
www.biorender.com

# Confirmation of Publication and Licensing Rights

**August 20th, 2026**

| | |
|---|---|
| ***Subscription Type:*** | *Student Plan - Academic* |
| ***Agreement number:*** | *VI2A3SX254* |
| ***Publisher Name:*** | *Advanced Heathcare Materials* |
| ***Citation to Use:*** | *Created in BioRender. Han, J. (2026) https://BioRender.com/8rqjjqe* |

To whom this may concern,

This document is to confirm that Jie Han has been granted a license to use the BioRender Content, including icons, templates, and other original artwork, appearing in the attached Completed Graphic pursuant to BioRender's Academic License Terms. This license permits BioRender Content to be sublicensed for use in publications (journals, textbooks, websites, etc.).

All rights and ownership of BioRender Content are reserved by BioRender. All Completed Graphics must be accompanied by the following citation: "Created in BioRender. Han, J. (2026) https://BioRender.com/8rqjjqe".

BioRender Content included in the Completed Graphic is not licensed for any commercial uses beyond use in a publication. For any commercial use of this figure, users may, if allowed, recreate it in BioRender under an Industry BioRender Plan.

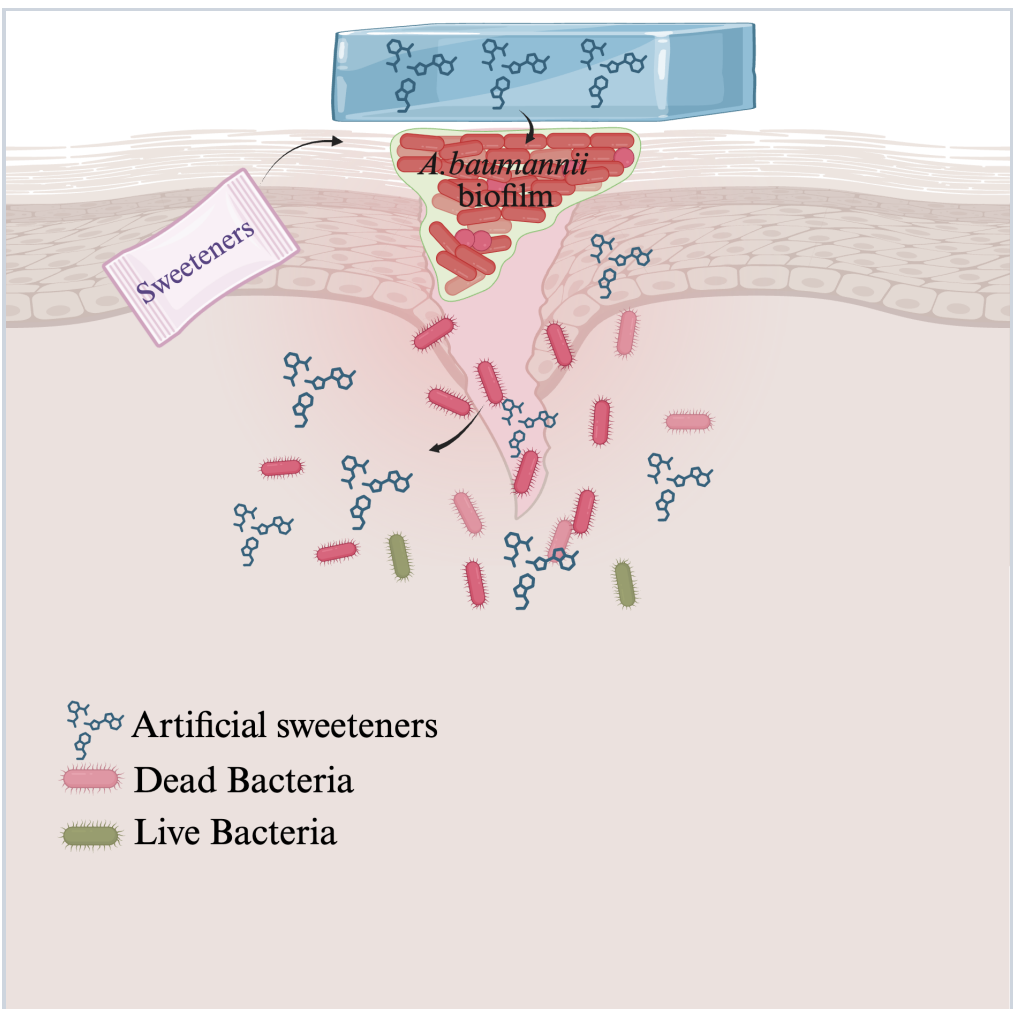


*For any questions regarding this document, or other questions about publishing with BioRender, please refer to our BioRender Publication Guide, or contact BioRender Support at support@biorender.com.*

**Figure 6S Publication License of Abstract Figure**

49 Spadina Ave. Suite 200
Toronto ON M5V 2J1 Canada
www.biorender.com

# Confirmation of Publication and Licensing Rights

**November 6th, 2025**

| | |
|---|---|
| ***Subscription Type:*** | *Student Plan - Academic* |
| ***Agreement number:*** | *RU28YRIEE0* |
| ***Publisher Name:*** | *Advanced healthcare materials* |
| ***Citation to Use:*** | *Created in BioRender. Han, J. (2025) https://BioRender.com/6sdwcby* |

To whom this may concern,

This document is to confirm that Jie Han has been granted a license to use the BioRender Content, including icons, templates, and other original artwork, appearing in the attached Completed Graphic pursuant to BioRender's Academic License Terms. This license permits BioRender Content to be sublicensed for use in publications (journals, textbooks, websites, etc.).

All rights and ownership of BioRender Content are reserved by BioRender. All Completed Graphics must be accompanied by the following citation: "Created in BioRender. Han, J. (2025) https://BioRender.com/6sdwcby".

BioRender Content included in the Completed Graphic is not licensed for any commercial uses beyond use in a publication. For any commercial use of this figure, users may, if allowed, recreate it in BioRender under an Industry BioRender Plan.

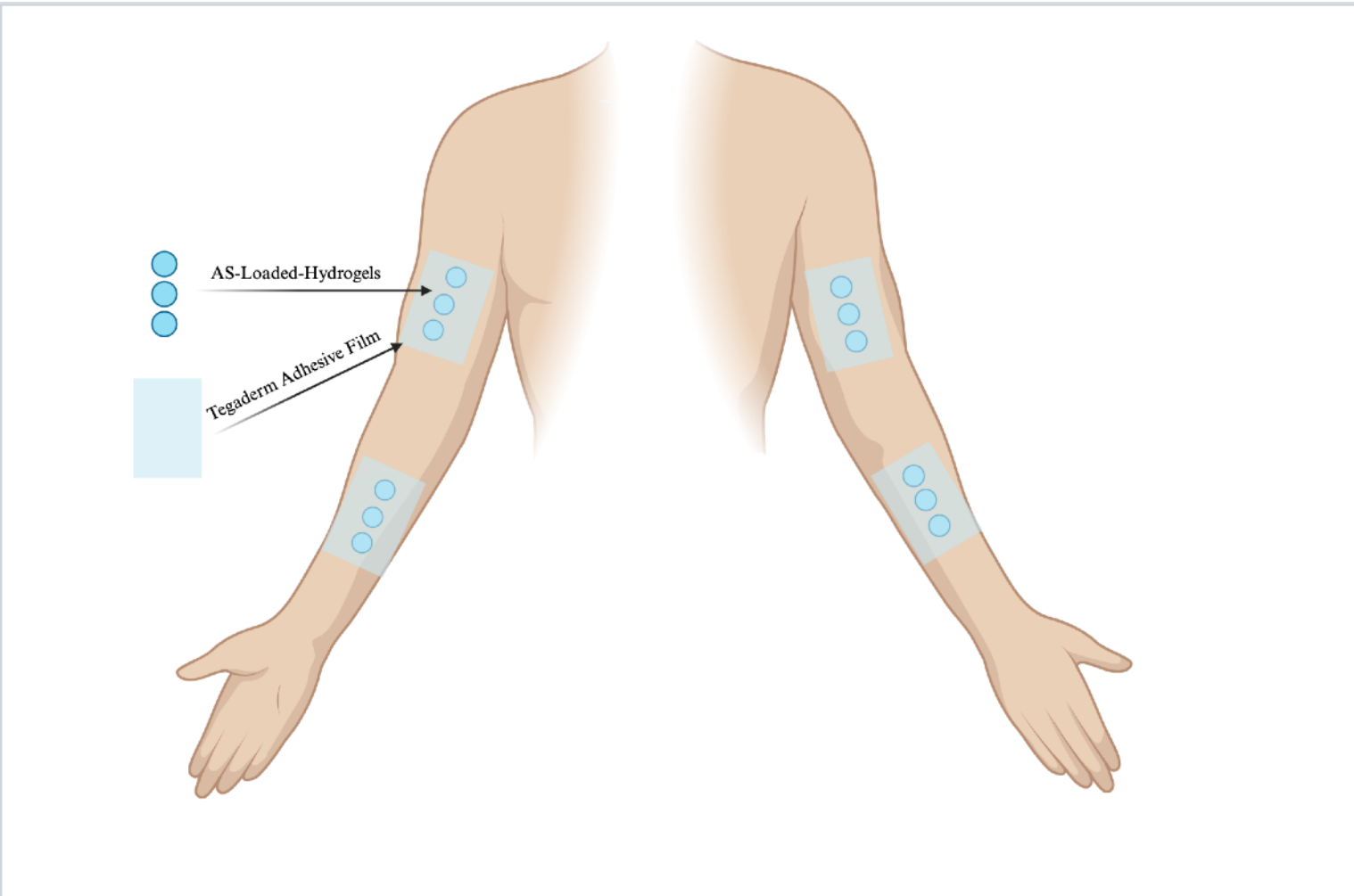


*For any questions regarding this document, or other questions about publishing with BioRender, please refer to our BioRender Publication Guide, or contact BioRender Support at support@biorender.com.*

**Figure 7S Publication License of Figure 11A**

49 Spadina Ave. Suite 200
Toronto ON M5V 2J1 Canada
www.biorender.com

# Confirmation of Publication and Licensing Rights

**June 6th, 2026**

| | |
|---|---|
| ***Subscription Type:*** | *Student Plan - Academic* |
| ***Agreement number:*** | *IZ29T1ECG5* |
| ***Publisher Name:*** | *Advanced healthcare materials* |
| ***Citation to Use:*** | *Created in BioRender. Han, J. (2026) https://BioRender.com/wurdw3w* |

To whom this may concern,

This document is to confirm that Jie Han has been granted a license to use the BioRender Content, including icons, templates, and other original artwork, appearing in the attached Completed Graphic pursuant to BioRender's Academic License Terms. This license permits BioRender Content to be sublicensed for use in publications (journals, textbooks, websites, etc.).

All rights and ownership of BioRender Content are reserved by BioRender. All Completed Graphics must be accompanied by the following citation: "Created in BioRender. Han, J. (2026) https://BioRender.com/wurdw3w".

BioRender Content included in the Completed Graphic is not licensed for any commercial uses beyond use in a publication. For any commercial use of this figure, users may, if allowed, recreate it in BioRender under an Industry BioRender Plan.

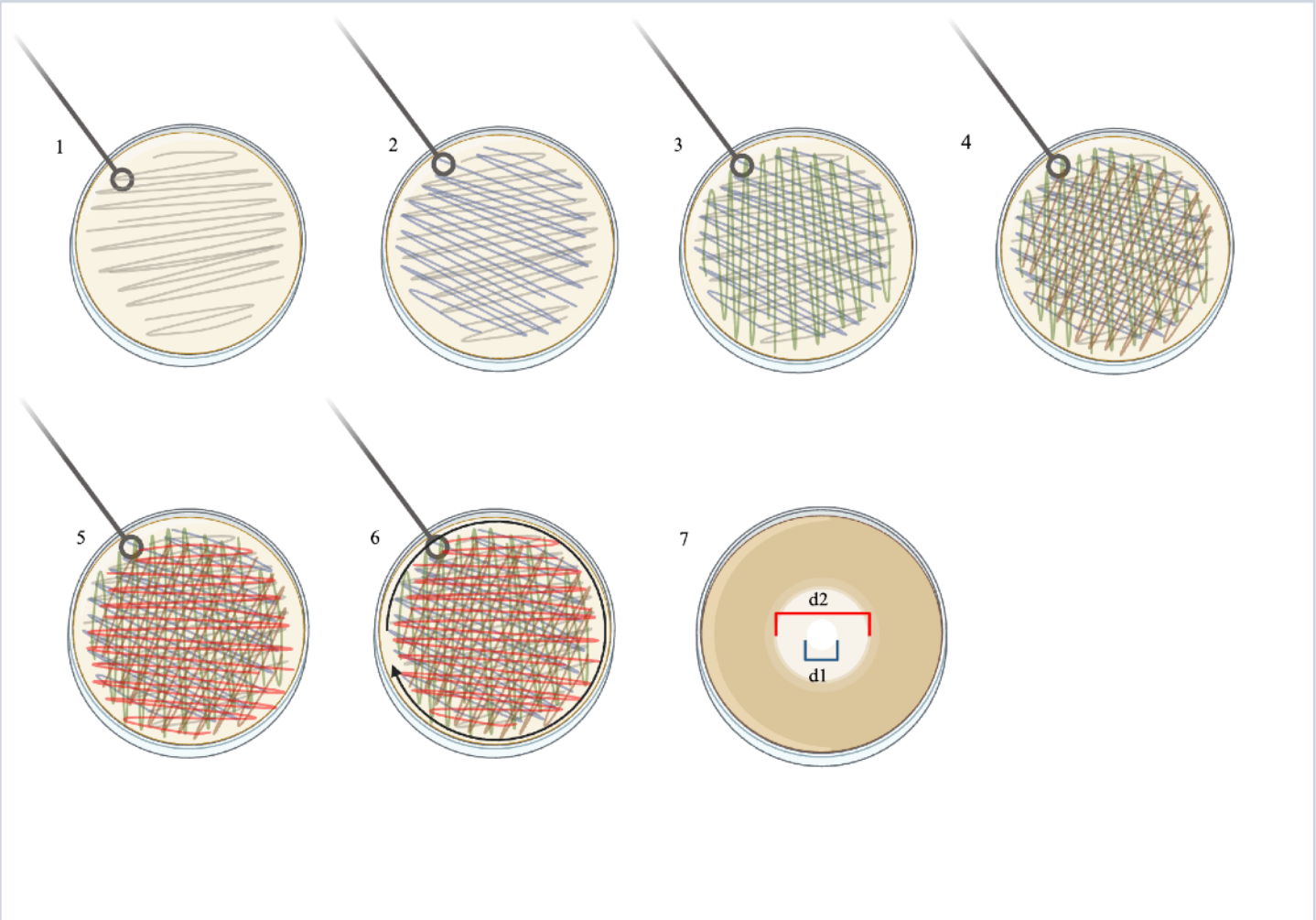


*For any questions regarding this document, or other questions about publishing with BioRender, please refer to our BioRender Publication Guide, or contact BioRender Support at support@biorender.com.*

**Figure 8S Publication License of Figure 1**
